# Metallo-coiled Coil Stabilization via Chemical Cross-Linking: Implications for Gd(III)-Based MRI Contrast Agents

**DOI:** 10.1021/jacs.5c13620

**Published:** 2025-11-05

**Authors:** Kate A. Hadley, Marco Ricci, Marko Hanzevacki, Helena Bernstein, Hiruni S. Jayasekera, Aneika C. Leney, Adrian J. Mulholland, Fabio Carniato, Mauro Botta, Melanie M. Britton, Anna F. A. Peacock

**Affiliations:** † School of Chemistry, 1724 University of Birmingham, Edgbaston B15 2TT, United Kingdom.; ‡ Dipartimento di Scienze e Innovazione Tecnologica, 325870 Università del Piemonte Orientale “A. Avogadro”, Alessandria 15121, Italy; § Centre for Computational Chemistry, School of Chemistry, 1980 University of Bristol, Bristol BS8 1TS, United Kingdom; ∥ School of Biosciences, University of Birmingham, Edgbaston B15 2TT, United Kingdom

## Abstract

Coiled coils are a versatile class of protein-inspired metal-binding ligands with well-defined sequence-to-structure relationships and high synthetic accessibility, making them promising tunable ligands for applications spanning biomedical imaging, catalysis, and material science. However, their practical utility is often limited by poor stability, particularly under physiological conditions. Herein, we introduce a covalent interhelical isopeptide cross-linking strategy that significantly enhances the thermal, thermodynamic, kinetic, and proteolytic stability of lanthanide-bound coiled coils designed for use as MRI contrast agents. Biophysical analysis, including CD, native MS, HPLC, and fluorescence assays, reveal that introduction of a single cross-linking layer promotes structural organization, driving the peptide from an unfolded to a well-folded state. Cross-linking leads to a remarkable 2.5 × 10^8^-fold enhancement in metal-binding affinity, along with greater kinetic stability and resistance to proteolytic degradation compared to the non-cross-linked analogue. ^1^H relaxometric studies and molecular dynamics simulations reveal that this class of potential MRI contrast agents operates via a second-sphere water coordination mechanism. Optimized cross-linking improves MRI efficacy by ∼30% at clinically relevant field strengths, highlighting its potential as a design principle for next-generation MRI contrast agents. Beyond MRI, these findings underscore the broader potential of covalent cross-linking strategies for enhancing the stability and functionality of metallo-coiled coils, expanding their utility across diverse applications.

## Graphical abstract

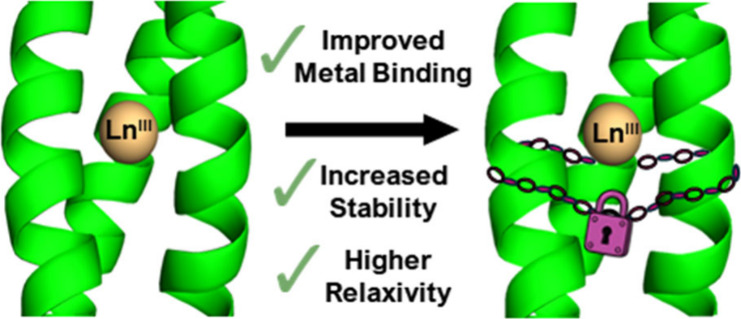

## Introduction

The chemistry of metals are essential across many fields, from catalysis^1^ to therapeutic applications.^2^ Ligands serve as essential tools for controlling and modulating the chemistry and overall performance of metal centers. While synthetic chemists typically rely on traditional small molecule ligands, nature has evolved protein ligands capable of generating metal-binding sites within 3D chiral environments rich in supramolecular chemistry. These protein scaffolds can modulate the chemistry of a bound metal ion in a way that can be difficult to replicate with small molecule ligands.^3^ However, natural proteins are often limited by their intrinsic complexity, stability issues, and their restriction to native amino acids and post translational modifications.

De novo designed miniature artificial protein scaffolds, such as coiled coils, offer many of the advantages of protein ligands, but without many of their limitations. The automation of solid phase peptide synthesis and the highly predictable sequence-to-structure relationships of coiled coil formation,^4,5^ offers great scalability, functionalization potential and design flexibility for use as metal ligands. Recent studies have shown that coiled coils provide a versatile ligand platform for designing metal-binding sites with high specificity and tunable properties,^6–9^ and in many cases, they outperform traditional small molecule ligands.^10–14^

We have previously shown that Gd­(III)-bound coiled coils, can exhibit superior relaxivity (*r*_1_ = 14.0 mM^–1^ s^–1^ and *r*_2_ = 32.7 mM^–1^ s^–1^ at 1.4 T),^11,14^ a measure of MRI contrast agent efficiency and performance, compared to clinical small-molecule Gd­(III)-based contrast agents such as Gd-DOTA (*r*_1_ = 4.3 mM^–1^ s^–1^ and *r*_2_ = 5.7 mM^–1^ s^–1^ at 1.5 T).^11^ These findings advance our understanding of ligand suitability in MRI contrast agent design and illustrate the potential of metallo-coiled coils to surpass the performance limits of traditional inorganic complexes. By exploiting the modular nature of the coiled coil scaffold, we have further tuned and enhanced the relaxivity performance of these systems.^15–17^ However, despite recent efforts to enhance stability through the use of a non-natural amino acid (norleucine),^18^ Gd­(III) coiled coils still face challenges in achieving the thermodynamic, kinetic and biological stability necessary to prevent the release of toxic Gd­(III), which remains a barrier for clinical translation.

One major barrier is the entropic penalty associated with assembling three individual peptide strands around a central metal ion. Various strategies have been explored to enhance coiled coil stability, including macrocyclization and terminal templating,^19^ as well as interhelical stapling.^20–25^ These approaches often involve replacing interhelical salt bridges with covalent linkages, such as amide bonds,^21^ triazoles,^20,23–25^ alkylated cysteine residues,^20^ or through activated furan warheads.^22^ Aupic et al. demonstrated that introducing a C-terminal disulfide bridge, designed to enforce coiled-coil orientation, also reduced the concentration dependence of folding and enhanced the thermal stability of the Zn^2+^ bound coiled-coil dimer complex. However, no quantitative data were reported regarding changes in metal-binding affinity or kinetic resilience.^26^ Despite these advances, including the work by Aupic et al., covalent cross-linking has not, to the best of our knowledge, been applied to directly stabilize metal coordination within the hydrophobic core of coiled coil assemblies.

Here, we show that introducing a covalent isopeptide cross-link adjacent to a destabilized Gd­(III)-binding site (see Figure 1) substantially enhances the thermal, thermodynamic, kinetic, and biological stability of the resulting Gd­(III) coiled coil complex, while also improving its performance as an MRI contrast agent. This level of stabilization is essential for advancing metallo-coiled coils toward clinical application.

**Figure 1:**
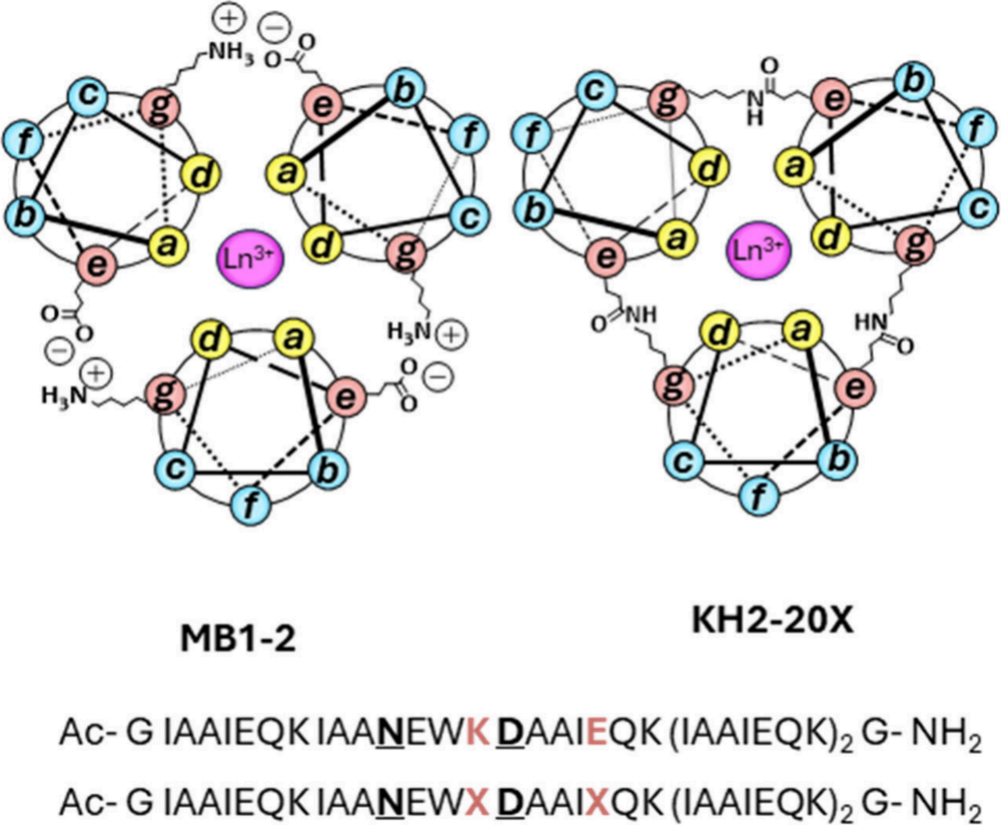
Helical wheel diagram depicting a cross-section of Ln­(MB1-2)_3_ and Ln­(KH2-20X), based on the heptad repeat (*abcdefg*)_*n*_. The peptide helices extend from the N-terminus (emerging from the page) to the C-terminus (projecting into the page). Interhelical salt bridges and isopeptide bonds across Glu (*e*) and Lys (*g*) positions are shown. Bound Ln­(III) ions are depicted as pink spheres. Also shown are the corresponding sequences for MB1-2 (top) and KH2-20X (bottom), where the binding site residues are bold and underlined and the salt bridge (K, E) or cross-link locations (X) are shown in bold orange.

## Results and Discussion

### Peptide Design

To address stability issues, a cross-linked analogue of our original design (MB1-2, a parallel three stranded coiled coil based on five I_*a*_A_*b*_A_*c*_I_*d*_E_*e*_Q_*f*_K_*g*_ heptad repeats) was developed. Here a layer of isopeptide interhelical cross-links was introduced adjacent to the destabilizing Asn_*d*_Asp_*a*_ Gd­(III) binding site, to yield KH2-20X (see Figure 1).^23^ In KH2-20X, the glutamic acid (Glu, E) at position 20 (*e* site) is modified to give rise to a thioester, for subsequent reaction with lysine (Lys, K) in a *g* site on an adjacent strand, to yield an interhelical isopeptide cross-link near the metal binding site, see Supporting Information Scheme S1. Cross-linking of the purified monomer (Figure S1) was performed with excess Tb­(III) to facilitate templated assembly and folding, helping preorganize the thioester and Lys. The reaction was monitored by analytical C18 reverse phase HPLC (Figure S1) and the fully cross-linked peptide trimer, KH2-20X, and its metal complexes, Tb/Gd­(KH2-20X), were characterized by ESI mass spectrometry (Figures S2 and S3 and Table S1). Tb­(III) and Gd­(III) are used interchangeably by us and others, due to their near identical coordination chemistry, but differing magnetic and emissive properties.

### Molecular Dynamics Simulations into Cross-Link Formation

To understand the structural impact of isopeptide cross-linking between Lys and Glu, molecular dynamic studies were carried out. Initial structural models were generated (Figures S4 and S5) and conformational flexibility was evaluated via root-mean-square deviation (RMSD) histograms (Figure S6), quantifying deviations from the AlphaFold2-predicted reference structure.^27^

Simulations were conducted for several systems: the non-cross-linked peptide, the fully cross-linked peptide between Glu_20_ and Lys_15_ (cross-link A, yielding the A_3_ structure), and peptides with alternative cross-links between Glu_20_ and Lys_22_ (cross-link B, yielding A_2_B, AB_2_ and B_3_ configurations). Among these, the B_3_ variant exhibited the largest RMSD values, indicating substantial structural instability.

To further investigate the impact of cross-linking on structural dynamics, principal component analysis (PCA) and free energy landscape (FEL) calculations were performed. All cross-linked Gd­(KH2-20X) systems displayed substantial overlap in conformational space with the non-cross-linked Gd­(MB1-2)_3_ system (Figure S7A). Notably, the A_3_ variant exhibited two distinct minima in its FEL, corresponding to two stable conformations (Figure S7B), suggesting enhanced structural stability and supporting its preferential formation in agreement with experimental findings (vide infra).

### Establishing the Cross-Link Location

To experimentally determine the positioning of the covalent cross-linker, enzymatic digestion of the peptide was carried out. Trypsin cleaves peptides at the C-terminal side of Lys and Arg residues, thereby facilitating peptide mapping and sequence analysis.^28^ Digestion of the MB1-2 peptide yielded four distinct peaks in the analytical C18 reverse phase HPLC, each corresponding to one of the unique heptad repeat sequences within the monomer strand (Figures S8A and S9 and Table S2). Although MB1-2 contains five total heptads, two are sequence-identical, resulting in only four distinguishable digestion products.

KH2-20X shares the same primary sequence as MB1-2, with the exception of a covalent isopeptide bond formed between a Lys and Glu residue. Therefore, its digestion pattern is expected to mirror that of MB1-2, except at the site of cross-linking. Consistent with this expectation, tryptic digestion of KH2-20X revealed two fragment peaks identical to those observed in MB1-2, along with a larger trimeric fragment corresponding to an uncleaved stretch encompassing two heptads, IAANEWK DAAIEQK (Figures S8B and S9 and Table S2). Additional trimeric fragments were detected, likely arising from incomplete cleavage near the N- or C-terminus. The absence of a trimeric fragment corresponding to the heptad DAAIEQK, combined with the absence of the individual IAANEWK and DAAIEQK fragments that were clearly observed in the MB1-2 digest, supports regioselective *e*′–*g* cross-link formation between Lys_15_ and Glu_20_.

### Cross-Linked Peptide Folding and Thermal Stability

Circular dichroism (CD) folding experiments highlight the significant difference in secondary structure between MB1-2 and its cross-linked analogue, KH2-20X. Apo MB1-2 is largely unstructured between 0.1 and 10 μM trimer (17 ± 1%). In contrast, KH2-20X has an α-helical structure across all concentrations studied (96 ± 3% at 10 μM); see Table 1 and Figures 2A,B and S10.

**Figure 2:**
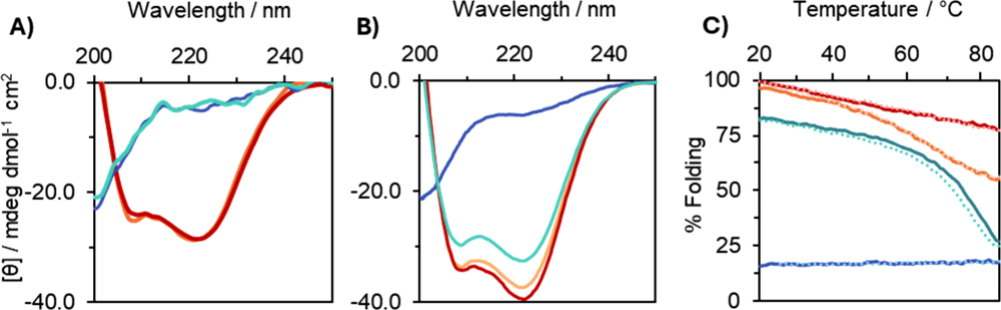
(A) CD spectra of 0.1 μM and (B) 10 μM KH2-20X and MB1-2 trimers in the absence (apo; KH2-20X, orange; MB1-2, blue) and presence (holo; KH2-20X + TbCl_3_, red; MB1-2 + TbCl_3_, turquoise) of 1.0 equiv of TbCl_3_, recorded at 293 K. (C) CD thermal unfolding profiles of 10 μM trimers monitoring the molar ellipticity at 222 nm: apo MB1-2 (blue), Tb­(MB1-2)_3_ (turquoise), apo KH2-20X (orange), and Tb­(KH2-20X) (red). Solid lines indicate forward heating, and dotted lines indicate reverse cooling. All experiments were conducted in HEPES buffer at pH 7.0, with buffer concentrations adjusted to match peptide concentration (0.1 and 10.0 mM for 0.1 and 10.0 μM peptide, respectively). For the 0.1 μM peptide samples, pH drifted between 6.8 and 7.3 due to the lower buffer capacity.

**Table 1:** Summary of Peptide Characterization Data: Association Constant (log *K*_a_); % Folding; Relaxivity at 60 and 20 MHz (^60^ *r*_1_, ^60^ *r*_2_, and ^20^ *r*_1_)

	Tb/Gd(MB1-2)_3_	Tb/Gd(KH2-20X)
log *K*_a_(Tb)	5.5 ± 0.2	13.9 ± 0.1
% apo folding (10.0 μM trimer)	17 ± 1	96 ± 3
% metalated folding, 1.0 equiv of TbCl_3_ (10.0 μM trimer)	84 ± 1	97 ± 2
^60^ *r*, _1_ mM^–1^ s^–1^	15.3 ± 0.6	19.6 ± 0.2
^60^ *r*_2_ mM^–1^ s^–1^	20.7 ± 0.1	37.4 ± 0.4
^20^ *r*_*1*_, mM^–1^ s^–1^	15.3	34.1

MB1-2 exhibits concentration-dependent folding behavior, driven by the assembly of three distinct peptide strands into a coiled coil (Figure 2A,B). Binding of Tb­(III) is known to template this assembly, promoting folding of the MB1-2 peptide trimer. At 1 and 10 μM (Figure 2B) trimer concentrations, Tb­(III) binding increases folding to 63 ± 4 and 84 ± 1%, respectively (Table 1). However, at 0.1 μM trimer, the addition of Tb­(III) fails to induce folding (Figures 2A and S10). Furthermore, the kinetics of metal binding and coiled coil assembly are similarly concentration dependent. Upon addition of 1.0 equiv of Tb­(III) to a 10 μM MB1-2 trimer solution, equilibrium is reached within approximately 40 min. This extends to several hours under more dilute conditions (1 μM MB1-2 trimer); see Figure S11A. In comparison, apo KH2-20X is already well-folded in the absence of metal, and Tb­(III) binding does not further enhance folding. Consequently, fluorescence spectroscopy monitoring the sensitized emission of bound Tb­(III) confirms that Tb­(III) binding to KH2-20X is rapid across all tested concentrations (0.1, 1.0, and 10 μM), consistent with a preorganized and structurally constrained metal-binding site (Figure S11B).

Thermal denaturation CD experiments were performed to investigate the impact of isopeptide cross-linking on coiled coil stability (Figure 2C). KH2-20X exhibits greater thermal stability than its non-cross-linked analogue MB1-2, consistent with prior observations in other cross-linked coiled coils.^21^ Although precise melting temperatures (*T*_m_) could not be determined due to the absence of well-defined unfolding transitions, Tb­(MB1-2)_3_ displays gradual thermal denaturation, decreasing from 82% folded at 20 °C to 26% at 85 °C. In contrast, both apo and Tb­(III)-bound KH2-20X remain well-folded throughout the entire temperature range, with Tb­(KH2-20X) retaining 77% of its folded structure at 85 °C. These results indicate a *T*_m_ exceeding 85 °C and highlight the significant stabilizing effect of the covalent isopeptide cross-link.

### Thermodynamic Stability

An initial indication of the enhanced thermodynamic stability of Ln­(III) binding to KH2-20X, compared to the non-cross-linked analogue MB1-2, is the significantly higher degree of metalation observed via native mass spectrometry. This preserves noncovalent interactions enabling coiled coil complexes to be detected.^29^ Under identical conditions, MB1-2 showed only 10–15% metalation (Figures 3A and S3), whereas KH2-20X exhibited near-complete metalation (95–100%, Figures 3B and S3).

**Figure 3:**
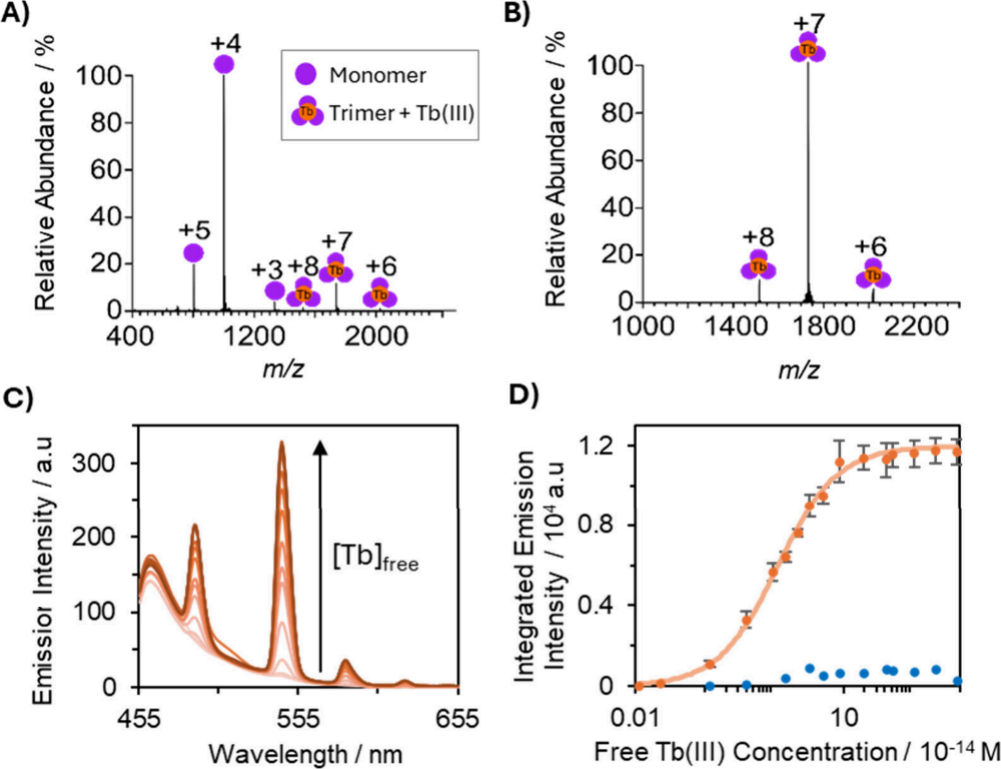
(A/B) Native mass spectrometry analyses of 10 μM (A) MB1-2 trimer and (B) KH2-20X with Tb­(III) in 50 mM ammonium acetate at pH 6.8. (C/D) Fluorescence spectra for the titration of 3.3 μM KH2-20X (orange) with Tb­(III)-EGTA in 50 mM HEPES buffer (pH 7.5, ionic strength of 0.1 M). (C) Representative full Tb­(III) emission spectra for each titration point. λ_ex_ = 280 nm. (D) Integrated emission intensity at 545 nm as a function of Tb­(III) concentration of 3.3 μM KH2-20X (orange) and MB1-2 trimer (blue). Error bars determined from standard deviation error of three independent repeat experiments. KH2-20X data were fitted to the Hills equation using MATLAB.

Previously, the log *K*_a_ for MB1-2 binding to Ln­(III) ions was determined by monitoring structural changes via CD. However, since KH2-20X does not undergo significant structural changes upon metal coordination, binding affinity was instead exclusively determined using fluorescence spectroscopy. Tb­(III) sensitized emission at 545 nm, associated with bound Tb­(III), was monitored as Tb­(III) was titrated into 10, 1, and 0.1 μM solutions of KH2-20X in 10 mM HEPES buffer pH 7.0. The resulting binding curves indicated a significantly stronger Tb­(III) affinity for KH2-20X compared to MB1-2 (Figure S12). In fact, the affinity was so high that a direct determination of log *K*_a_ was not possible under these conditions.

Consequently, competition experiments were performed using ethylene glycol–bis­(β-aminoethyl ether)-*N*,*N*,*N*′,*N*′-tetraacetic acid (EGTA), following the protocol outlined by Cotruvo et al.^30,31^ These studies yielded an apparent log *K*_a_ of 13.9 ± 0.1 for Tb­(KH2-20X) (Figure 3C,D), in stark contrast to the previously reported value of 5.5 ± 0.2 for MB1-2. Parallel EGTA competition studies with MB1-2 showed that the integrated Tb­(III) emission closely matched that of the Tb­(EGTA) complex, indicating near-complete displacement of Tb­(III) and negligible binding to MB1-2 under these conditions. As a result, binding constants for MB1-2 could not be determined using this method. This large difference in binding affinity underscores the substantial thermodynamic stabilization conferred by covalent cross-linking in KH2-20X.

### Kinetic Stability

Chemical cross-linking has been shown to significantly enhance the thermodynamic stability of the system. However, whether this modification also confers improved kinetic stability, a critical parameter for clinical translation, required further investigation.^32,33^ To evaluate this, the kinetic robustness of the Tb­(III) complexes of KH2-20X was assessed against biologically relevant competing ligands (phosphate) and metal ions (Zn­(II)).

Phosphate, a known chelator of lanthanides, is present in human blood at concentrations ranging from 1 to 1.5 mM (3.0–4.5 mg/dL, phosphorus).^34^ Displacement experiments were carried out to simulate physiologically relevant conditions, wherein 1.2 mM phosphate was added to solutions of Tb­(MB1-2)_3_ and Tb­(KH2-20X), and Tb­(III) emission at 545 nm was monitored over time. A decline in emission intensity would indicate displacement of Tb­(III) due to phosphate coordination. As expected, Tb­(MB1-2)_3_ exhibited poor kinetic stability, with complete loss of Tb­(III) observed within 1.5 h. In contrast, Tb­(KH2-20X) retained approximately 90% of the emission intensity (compared to a HEPES control) after 22 h, demonstrating a markedly higher resistance to phosphate-induced dissociation (Figure S13).

Given that Zn­(II) is present in blood at concentrations of 55–125 μM,^35^ its ability to promote transmetalation was also assessed. Conventionally, the kinetic stability of Gd­(III) complexes against Zn­(II)-mediated transmetalation, is evaluated by monitoring relaxivity changes in phosphate buffer, as displaced Gd­(III) forms insoluble GdPO_4(s)_.^36,37^ However, due to the inherent instability of Gd­(MB1-2)_3_ in phosphate, this approach was unsuitable. Instead, transmetalation of the Tb­(III) complexes was assessed by monitoring changes in Tb­(III) emission intensity following incubation with 125 μM Zn­(II). Interestingly, no significant change in Tb­(III) emission was observed for either Tb­(MB1-2)_3_ or Tb­(KH2-20X) over time (Figure S13), indicating that both complexes possess highly selective metal-binding sites^38^ with strong resistance to Zn­(II)-induced transmetalation, reflecting the kinetic stability of these Tb­(III) complexes.

The stability of MRI contrast agents is often benchmarked by the “ratio index”, defined as the time for the relaxation value to drop to 80% of its initial value under Zn­(II)/phosphate challenge. Magnevist, a linear agent, exhibits a ratio index of ∼250 min, while Dotarem, a macrocyclic agent, exceeds 5000 min.^37^ In comparison, the cross-linked Tb­(KH2-20X) complex demonstrated a ratio index of ∼1320 min, substantially outperforming the non-cross-linked Tb­(MB1-2)_3_ trimer (∼1 min) and surpassing many linear agents. Laurent et al. monitored complex integrity using relaxivity under equimolar conditions (2.5 mM complex and 2.5 mM Zn­(II)).^36^ In contrast, our assays employed Tb­(III) fluorescence, which provides a direct and sensitive readout of complex stability, using ∼1 μM complex challenged with 125 μM Zn­(II), placing Zn­(II) in significant excess. Both complexes remained stable under Zn­(II) challenge; however, phosphate induced more rapid displacement of Tb­(III) (see Figure S13), and the reported ratio indices reflect this phosphate-driven dissociation.

Taken together, these findings highlight that cross-linking significantly enhances kinetic robustness. With further optimization, peptide-based scaffolds may approach, or even rival, the stability of established macrocyclic agents.

### Proteolytic Stability

Apo and Tb­(III)-bound forms of MB1-2 and KH2-20X were subjected to proteolytic degradation using 12 μM trypsin, a broad-specificity protease that cleaves at Lys and Arg residues. Trypsin was chosen to (1) validate cross-link positioning and (2) assess the proteolytic stability of this new class of cross-linked metallo-coiled coils for potential in vivo applications. Apo MB1-2 was rapidly degraded within 30 min (Figures 4 and S14), yielding peptide fragments consistent with cleavage at all Lys sites (Figures S8A, S9, and S14, and Table S2.

**Figure 4:**
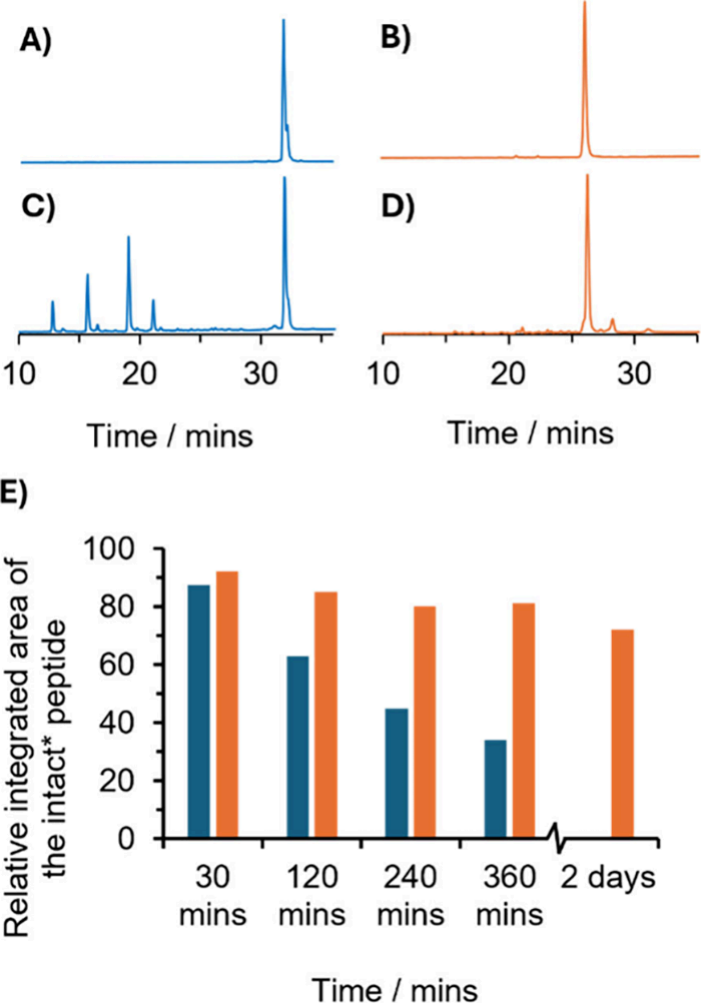
Analytical C18 reverse phase HPLC spectra of 100 μM Tb­(MB1-2)_3_ (A, C) and Tb­(KH2-20X) (B, D) after 6 h of incubation in the absence (A, B) and presence (C, D) of 12 μM trypsin in 100 mM HEPES buffer (pH 7.5). A linear H_2_O/MeCN gradient (0–50% MeCN over 40 min) in the presence of 0.05% TFA, was applied. (E) Relative integrated area of intact MB1-2* and KH2-20X* as a function of time, where * denotes MB1-2 or KH2-20X lacking the C-terminal G-NH_2_ group.

In contrast, apo KH2-20X exhibited significantly enhanced proteolytic stability. After 30 min of trypsin exposure, the predominant species (∼74%) remained intact as trimers, either lacking the C-terminal G-NH_2_ group (main species) or the Ac-G IAAIEQK fragment (Figure 4 and Figures S8B, S9, and S15 and Table S2). This population gradually declined to ∼45% after 6 h. Meanwhile, the abundance of a peak corresponding to the trimeric cross-linked IAANEWK DAAIEQK fragment increased from 5% at 30 min to 20% at 6 h, consistent with the cross-linked Lys_15_ being resistant to enzymatic cleavage. Comparable improvements in proteolytic resistance have been observed in other cross-linked coiled coil systems.^21^

While Tb­(MB1-2)_3_ showed moderate protection from degradation relative to its apo form, over 50% of the complex was degraded within 4 h, and no intact species remained after 48 h. In contrast, Tb­(KH2-20X) retained 72% of the intact trimer after 48 h of incubation. The primary degradation product involved loss of the C-terminal G-NH_2_ residue (yielding MB1-2* and KH2-20X*; Figure 4 and Figure S9 and Table S2) and is unlikely to significantly impact functional properties. The next most abundant degradation product lacked both the C-terminal G-NH_2_ group and the N-terminal Ac-G IAAIEQK heptad, while retaining the covalent cross-link and the metal-binding site. This suggests that key structural and functional elements of the peptide remain preserved despite partial proteolysis.

### MRI Performance Enhancement

To assess the impact of covalent isopeptide cross-linking on MRI efficacy, the longitudinal (*r*_1_) and transverse (*r*_2_) relaxivities of Gd­(KH2-20X) were measured. The cross-link not only preserves MRI performance but also enhances relaxivity, with an approximate 30% increase in *r*_1_ at clinically relevant field strengths (60 MHz) (Figure S16 and Table 1).

To elucidate the mechanism underlying this enhancement and to better understand contrast generation in this class of Gd­(III) coiled coil complexes, we conducted ^1^H nuclear magnetic relaxation dispersion (NMRD) and molecular dynamics simulations. The absence of inner-sphere water molecules in Tb­(MB1-2)_3_ suggests that relaxivity in these systems primarily arises from outer-sphere and second-sphere mechanisms.^11,15^ Consistent with this, ^1^H NMRD studies and analysis yielded the best fit for both Gd­(MB1-2)_3_ and Gd­(KH2-20X) when a significant second-sphere contribution was included. The optimal model accounts for two second-sphere water molecules (*q* ^ss^) positioned at a distance (*r* ^ss^, H_wat_-Gd­(III)) of 3.6 Å from the Gd­(III), undergoing fast exchange with bulk water molecules (Figures 5 and S17–S19).

**Figure 5:**
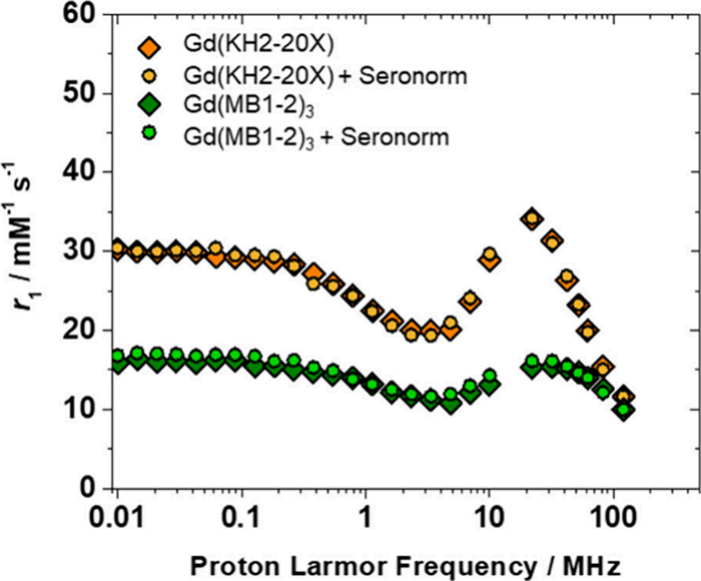
Comparison of ^1^H NMRD profiles of Gd­(MB1-2)_3_ (green) ([Gd^3+^)] = 0.4 mM) and Gd­(KH2-20X) (orange) ([Gd^3+^)] = 0.3 mM) in HEPES buffer (◊) and in seronorm (○), at 298 K and pH 7.4. The seronorm solution was prepared by dissolving 90 mg of the biological matrix in 1 mL of peptide aqueous solution.

The model further suggests the presence of two distinct types of motion: a relatively fast local rotation of second-sphere water molecules hydrogen-bonded to polar groups near the Gd­(III) (τ_RL_) and the slower global motion of the overall system (τ_RG_).^39,40^ The correlation between these motions is quantified by the order parameter (*S* ^2^), which ranges from zero (independent motions) to one (fully correlated motions). To refine the analysis key parameters were fixed: τ_RG_ (global rotational correlation time, set at 5 ns for Gd­(MB1-2)_3_ and 7 ns for Gd­(KH2-20X)), *a* (the distance of closest approach for outer-sphere water molecules, set at 4 Å), and *D* (the relative diffusion coefficient, set at 2.3 × 10^–10^ m^2^ s^–1^) (Table S3). Meanwhile, τ_RL_ (local rotational correlation time of second-sphere water), τ_M_^SS^ (mean residence time of second-sphere water), and *S* ^2^ were treated as adjustable parameters (Table S3). The fitted τ_RL_ values reveal that second-sphere water molecules interacting with Gd­(III) in the cross-linked trimer Gd­(KH2-20X) exhibit lower rotational mobility and longer residency time (τ_RL_ = 318 ps; τ_M_^SS^ = 5.0 ns) than those in the non-cross-linked Gd­(MB1-2)_3_ (τ_RL_ = 184 ps; τ_M_^SS^ = 3.0 ns). This more constrained second-sphere water dynamics correlates with the increase in relaxivity, highlighting the functional benefits of covalent cross-linking in stabilizing second-sphere water interactions near the Gd­(III) center.^41^

### Molecular Dynamics Simulations into Gd­(III) Water Distribution

Molecular dynamics (MD) simulations were performed to gain molecular-level insight into water coordination dynamics. Radial distribution function (RDF) analyses revealed significant differences in water distribution around the Gd­(III) metal across systems (see Figure 6 for the comparison between the non-cross-linked Gd­(MB1-2)_3_ and the cross-linked Gd­(KH2-20X) A_3_ system, and Figures S20–S22 for RDFs of all investigated systems). In the A_3_ cross-linked variant, water molecules were more frequently observed within the second coordination sphere (H_Wat_-Gd­(III) ≈ 3.6 Å) compared to both the non-cross-linked and B_3_-cross-linked systems. Mixed cross-linking configurations (A_2_B, AB_2_) exhibited intermediate RDF profiles, consistent with the extent of A or B cross-linking.

**Figure 6:**
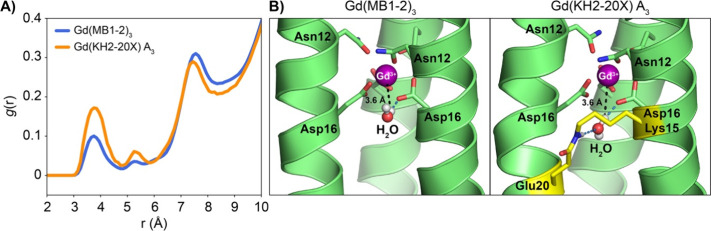
(A) Radial distribution function (RDF) of water hydrogen atoms (H_Wat_) relative to Gd­(III) in the non-cross-linked Gd­(MB1-2)_3_ system and the cross-linked Gd­(KH2-20X) A_3_ variant. (B) Representative MD snapshots of both systems, highlighting second-sphere water molecules within the cavity near the Gd­(III) ion (depicted as a magenta sphere). The peptide cross-link is indicated in yellow.

Water occupancy in the Gd­(III) second coordination sphere was systematically analyzed during MD simulations (Figure S23). In the non-cross-linked Gd­(MB1-2)_3_ system, the average occupancy was ∼1 water molecule, with the region devoid of water ∼45% of the time and occupied by a single molecule ∼35% of the time. In contrast, the cross-linked Gd­(KH2-20X) A_3_ system showed a higher average occupancy (∼1.5), with water-free occupancy reduced to ∼10% and the region occupied by one or two molecules ∼45 and ∼40% of the time, respectively. These results indicate that cross-linking across A_3_ enhances the second-sphere hydration level around the metal site.

Second-sphere water lifetime analysis indicated that longer residence times (∼4 ns) were more prevalent in the A_3_ cross-linked Gd­(KH2-20X) system relative to the non-cross-linked Gd­(MB1-2)_3_ (∼2 ns) (Figure 6). This trend aligns with the values obtained from ^1^H NMRD analysis, reinforcing the role of cross-linking in stabilizing second-sphere water molecules.

MD snapshots further illustrate that the A cross-link is positioned directly adjacent to the Gd­(III) binding site, where hydrogen bonding interactions involving the amide cross-link promote the local preorganization of second-sphere water molecules. In contrast, the B cross-link is spatially distant from the Gd­(III) site and does not significantly influence water positioning in the second coordination sphere.

### MRI Performance in a Biological Matrix

The incorporation of a covalent cross-linking layer significantly enhances the thermodynamic, kinetic, and proteolytic stability of the system, while not only preserving but, when positioned adjacent to the Gd­(III) site, enhances MRI relaxivity. However, the ability of these agents to maintain performance in a complex biological matrix, such as human serum, remains a critical consideration for clinical translation.

To evaluate this, ^1^H NMRD profiles of Gd­(III)-coordinated MB1-2 and KH2-20X were measured in Seronorm (a human serum matrix), providing insights into potential interactions with endogenous biomolecules. Dissociation of Gd­(III) from the peptide scaffold would manifest as pronounced changes in relaxometric properties due to differences in metal ion hydration states across distinct environments. Additionally, nonspecific binding to serum proteins could alter molecular tumbling dynamics, particularly affecting relaxivity at high magnetic fields.

For both Gd­(MB1-2)_3_ and Gd­(KH2-20X), ^1^H NMRD profiles measured in seronorm closely match those obtained in aqueous solution (Figure 5 and Table S4), indicating effective Gd­(III) retention and a lack of significant interactions with serum proteins. This result underscores the inertness of the system under biologically relevant conditions.

Taken together, the preserved relaxometric properties in serum, combined with the demonstrated thermodynamic, kinetic, and proteolytic stability conferred by covalent cross-linking, highlight Gd­(KH2-20X) as a promising candidate for in vivo imaging applications.

## Conclusions

The development of stable, well-defined and robust metallo-coiled coils is critical to unlocking their full potential across a broad range of applications. In this study, we introduce a covalent isopeptide cross-linking strategy that substantially enhances the structural and functional stability of Gd­(III)-based metallo-coiled coils and demonstrates its utility in the design of high-performance MRI contrast agents.

Covalent interhelical cross-linking emerges as a powerful structural reinforcement tool, conferring significant improvements in thermal, thermodynamic, kinetic, and proteolytic stability. This stabilization is particularly vital for biomedical applications that demand prolonged structural integrity, such as with MRI contrast agents. Notably, the identity and positioning of the cross-link plays a pivotal role in modulating metal–ligand interactions beyond the first coordination sphere, directly influencing MRI contrast efficacy.

Relaxometric analyses combined with molecular dynamics simulations reveal that contrast generation in these Gd­(III) coiled coil systems arises primarily from a second-sphere water relaxation mechanism. This mechanism is retained in complex biological media, including human serum, highlighting the bioinertness and structural resilience of the design. Furthermore, strategic selection of cross-link identity and positioning results in a ∼30% increase in relaxivity at clinically relevant field strengths, further enhancing MRI performance. These findings position covalent cross-linking as a pivotal advancement in the design of next-generation, peptide-based MRI contrast agents.

Beyond MRI, the ability to enhance stability and exert precise control over the second coordination sphere through covalent cross-linking, presents opportunities for broader applications in catalysis,^42^ sensing and materials science. We anticipate that covalent cross-linking represents a critical design advancement for metallo-coiled coils, addressing key challenges in functional biomolecular engineering. This work establishes a foundation for the continued development of these systems, offering a versatile and tunable platform for real-world applications requiring high stability and well-defined coordination environments.

## Supplementary Material



## Notes

Materials and methods, peptide characterization, and supporting experimental HPLC, MS, CD, NMRD, and MD results cited in this work (PDF)
