# Supplementary material for: Metallo-coiled Coil Stabilization via Chemical Cross-Linking: Implications for Gd(III)-Based MRI Contrast Agents
Source: J Am Chem Soc. 2025 Nov 5;147(46):42583–90. doi: 10.1021/jacs.5c13620 (PMC12636026; doi:10.1021/jacs.5c13620)
Supplement: Supplementary file 1 [file ja5c13620_si_001.pdf]

Supporting Information

Metallo-coiled coil stabilization via chemical cross-linking: Implications for Gd(III)-based MRI contrast agents

Kate A. Hadley,[a] Marco Ricci,[b] Marko Hanzevacki,[c] Helena Bernstein,[d] Hiruni S. Jayasekera,[d] Aneika C. Leney,[d] Adrian J. Mulholland,[c] Fabio Carniato,[b] Mauro Botta,[b] Melanie M. Britton,[a] Anna F. A. Peacock*[a]

[a.] *School of Chemistry, University of Birmingham, Edgbaston, B15 2TT, UK*

[b.] *Dipartimento di Scienze e InnovazioneTecnologica, Università del Piemonte Orientale "A. Avogadro", Alessandria 15121, Italy*

[c.] *Centre for Computational Chemistry, School of Chemistry, University of Bristol, Bristol BS8 1TS, UK*

[d]*School of Biosciences, University of Birmingham, Edgbaston, B15 2TT, UK*

*To whom correspondence should be addressed. E-mail: a.f.a.peacock@bham.ac.uk

Contents

Materials and Methodology 3
Scheme S1: Reaction scheme of KH2-20X 11
Figure S1: HPLC and MS monitoring of KH2-20X cross-linking reaction 12
Figure S2: Analytical HPLC and MS characterization of KH2-20X monomer and trimer 13
Figure S3: Native mass spectrometry of Tb/Gd(III) metalated MB1-2 and KH2-20X 14
Table S1: The average theoretical and observed masses of MB1-2 and KH2-20X 15
Figure S4: Protocol for molecular dynamics studies: Non-cross-linked Gd(MB1-2)$_3$ 16
Figure S5: Protocol for molecular dynamics studies: Cross-linked Gd(KH2-20X) 17
Figure S6: RMSD calculations for Gd(MB1-2)$_3$ and Gd(KH2-20X) 18
Figure S7: Free energy landscape calculations for Gd(MB1-2)$_3$ and Gd(KH2-20X) 19
Figure S8: Peptide sequencing by trypsin degradation 20
Figure S9: Mass Spectra of peptide fragments from MB1-2 and KH2-20X trypsin degradation 21
Table S2: The theoretical and observed mass of peptide fragments from the degradation of KH2-20X and MB1-2 in trypsin and their corresponding sequences 22
Figure S10: Circular dichroism folding data 23
Figure S11: CD kinetic data MB1-2 and KH2-20X 24
Figure S12: Fluorescence Tb(III) binding titrations of MB1-2 and KH2-20X 25
Figure S13: Phosphate and Zn(II) kinetic stability studies 26
Figure S14: MB1-2 degradation studies with trypsin 27
Figure S15: KH2-20X degradation studies with trypsin 28
Figure S16: Relaxivity data for Gd(KH2-20X) and Gd(MB1-2)$_3$ 29
Figure S17: ^{1}H NMRD profiles of Gd(MB1-2)$_3$ and Gd(KH2-20X) 30
Figure S18: 62 MHz r_1 relaxivity temperature dependence studies 31
Figure S19: ^{1}H NMRD r_2 relaxivity studies 32
Table S3: Best fit parameters from the analysis of ^{1}H NMRD data 33
Figure S20 Radial distribution function of water in the SS for Gd(MB1-2)$_3$ and Gd(KH2-20X) 34
Figure S21: Radial Distribution Functions of water hydrogen atoms from Gd(III) 35
Figure S22: Comparison of second-sphere water probability 36
Figure S23 Average water molecules in the SS for Gd(MB1-2)$_3$ and Gd(KH2-20X) 37
Table S4: Longitudinal (r_1) and transverse (r_2) relaxivities for Gd(MB1-2)$_3$ and Gd(KH2-20X) at 60 MHz and 298 K, in the absence and presence of 0.6 mM Human Serum Albumin (HSA). 38

Materials and Methodology

Peptide Synthesis and Purification: Fmoc protected amino acids were purchased from Sigma Aldrich, AGTC Bioproducts, Cambridge Reagents or Novabiochem. Peptides were synthesized on either a CEM Liberty Blue or a PurePep Chorus peptide synthesizer on Rink Amide MBHA resin (0.25 mmol scale) using standard deprotection (20% piperidine in DMF). For synthesizes carried out on the CEM liberty blue peptide synthesizer, standard HBTU/HoBt coupling conditions were used with 0.1 M HoBt in the deprotection mixture.[1] For synthesizes carried out on the PurePep Chorus, couplings were performed using DIC (5 equiv) and Oxyma Pure (5 equiv) in DMF as coupling agents with 0.1 % formic acid in the deprotection mixture. Upon completion, all peptides were capped, and in the case of MB1-2, purified and characterized as previously reported.[2] The sequence for MB1-2 is Ac-G IAAIEQK IAANEWK DAAIEQK IAAIEQK IAAIEQK G-NH_2. KH2,20X has the same sequence as MB1-2 except the glutamic acid in position 20, where a glutamic acid with an alloc protecting group (X) instead of the standard ^{t}Bu protecting group, was used. The sequence for KH2-20X is Ac-G IAAIEQK IAANEWK DAAIXQK IAAIEQK IAAIEQK G-NH_2.

Synthesis and purification of KH2-20X monomer: Deprotection of the glutamic acid alloc protecting group was carried out on resin, using 1.0 equiv tetrakistriphenylphoshine, 10.0 equiv of dimedone in 50:50 dry DCM and THF under a N_2 atmosphere at r.t for two hours. The resin was rinsed with 3 × 7 ml 0.5% DIEA in DMF, 3 × 7 ml of 1 M sodium diethyldithiocarbamate in DMF, and 3 × 7 ml of Et_2O. The resin was left to air dry for 60 min. For the formation of the thioester, the peptide (on resin) was reacted with 5.0 equiv of benzyl mercaptan, 5.0 equiv of HBTU and 3.0 equiv of DIEA in DMF. The reaction was stirred at r.t over night. The peptide was then cleaved from the resin using standard procedures.[1] The peptide monomer was then purified as previously reported.[2]

Synthesis and purification of KH2-20X trimer: Pure KH2-20X thioester monomer was reacted at 40°C in 50:50 H_2O:MeCN, with excess Tb(III) (~ 5 .eq per trimer) buffered in 200 mM HEPES (pH 9) over 3 days. The reaction was modified from the procedure described by Wang and coworkers.[3,4] The reaction progression was monitored by analytical reverse phase C18-HPLC. Upon full conversion, which typically takes around 3 days, ~50 equiv of EDTA was added to remove bound Tb(III). The solution was left at r.t for around 60 min. before the solution was filtered and the cross-linked peptide purified by preparative reverse phase C18-HPLC. A conservative estimate being that >75% is recovered from cross-linking

pure KH2-20X thioester monomer. Upon successful purification, removal of Tb(III) was confirmed on a small aliquot of cross-linked peptide by fluorescence spectroscopy. Here the absence of enhanced/sensitized Tb(III) emission, at 545 nm, is consistent with complete removal of Tb(III) from the KH2-20X binding site.

Stock solutions preparation: In all experiments, the concentration of peptide stock solutions was determined, in triplicate, via the tryptophan absorbance ($\varepsilon_{280\ nm}$ = 5690 M^{-1} cm $^{-1}$) at 280 nm in water. Once samples were prepared, the concentration of the solutions were checked by UV vis and adjust if necessary. All $GdCl_3$ and $TbCl_3$ stock solutions were freshly prepared (~1 mM) in MilliQ water and their concentration was determined in triplicate using EDTA and xylenol orange indicator as previously reported by Fedeli *et al.*[5] All $ZnCl_2$ solutions were also freshly prepared and determined in a similar fashion using EDTA and xylenol orange indicator solution.[6] The equivalence of metal added was then adjust to the exact concentration of peptide in the sample. To prepare 0.1 mM stock solutions of $TbCl_3$ and $GdCl_3$, a 1 mM sample was prepared by the titration method above and diluted 10-fold in volumetric glassware using MilliQ water.

Native mass spectrometry (MS): Lyophilized MB1-2 and KH2-20X were diluted into 50 mM ammonium acetate pH 6.8. The concentrations of the peptides were measured using the absorbance at 280 nm assuming an extinction co-efficient of 5690 $M^{-1}cm^{-1}$ and 17070 $M^{-1}cm^{-1}$ for MB1-2 and KH2-20X, respectively. Metal stock solutions were initially prepared by dissolving in MilliQ water to a concentration of 10 mM. These stock solutions were then diluted into 50 mM ammonium acetate pH 6.8 immediately before use.

For native MS, MB1-2 and KH2-20X were analyzed at a final concentration of 10 µM. Each peptide was incubated with metals (Gd(III) and Tb(III)) at an equimolar amount of peptide to metal ratio of 10 µM:10 µM, assuming MB1-2 is a trimer. All samples were incubated at room temperature for 60 min. before analysis.

All native mass spectrometry measurements were performed using a Q-Exactive HF mass spectrometer (Thermo Fisher Scientific). Borosilicate needles were pulled in-house using a P-1000 micropipette puller (Sutter Instrument, Novato, CA) and were gold coated. Nano-electrospray ionization was performed in positive ion mode with key settings including 1.0–1.2 kV capillary voltage, capillary temperature 250 °C, S-lens 60, *m/z* range 400–2500, AGC target 1e6, maximum injection time 10 ms, and resolution 60,000 at 200 *m/z*. Spectra were acquired for 3 min., and the data were processed using XCalibur v.4.1 software (Thermo

Fisher Scientific). The observed molecular weights were calculated from an average of 3 charge states. For Gd- and Tb-bound complexes, both the metal (3+ charge) and H^+ contributed to the protonation states observed in the mass spectrum. For example, a 7+ MB1-2-Gd complex ion comprised of 3+ charges from the metal and 4 H^+s $(Gd^{3+} + 4H^+)^{7+}$.

NMR relaxation experiments: ^1H NMR relaxation experiments were carried out and analyzed as previously reported.[7] The T_1 and T_2 relaxation times were measured for solutions of $GdCl_3$ concentrations (0, 10, 20, 30, 40 μM) in the presence of 5 equivalence of peptide trimer buffered at pH 7.0 by 100 mM HEPES. Samples were left to equilibrate for an 60 min. before measurements were recorded. r_1 and r_2 relaxivities were calculated from the gradient of $1/T_1$ and $1/T_2$ as a function of $GdCl_3$ concentration respectively and reported from the average of 3 independent repeats.

T_1 and T_2 relaxation times were also recorded for KH2-20X and MB1-2 in the presence of 0.6 mM lyophilized human serum albumin buffered in 100 mM HEPES at pH 7.0. Here the peptide was left to equilibrate for 60 min. with $GdCl_3$ before the human serum albumin was added. Once the HSA was added, the samples were left for a further 60 min. to equilibrate before the T_1 and T_2 experiments were conducted.

^{1}H NMRD, preparation of Gd(MB1-2)$_3$ and Gd(KH2-20X): To a 6.3 mM MB1-2 monomer solution in 770 μL, 55 mg of HEPES were added to achieve a 300 mM buffer concentration. The pH was adjusted to 7.0 by gradually adding 0.5 M NaOH. Subsequently, 32 μL of the $GdCl_3 \cdot 6H_2O$ stock solution (in a 1:15 molar ratio to the peptide) was introduced into the peptide-buffered solution. No pH variation was observed after the addition. The concentration of Gd(III) in the final sample volume was estimated to be 0.40 mM, a determination further validated by the BMS method.[8]

^{1}H NMRD characterization: The magnetic-field dependence of the longitudinal relaxation rate (R_1) of solvent protons (^1H NMRD profiles) were measured in aqueous solution using a variable field relaxometer equipped with an HTS-110 3T Metrology Cryogen-free Superconducting Magnet (Mede, Italy), operating in the overall range of proton Larmor frequencies of 20-120 MHz (0.47-3.00 T). The measurements were performed using the standard inversion recovery sequence (20 experiments, 2 scans) with a typical 90° pulse width of 3.5 μs and the reproducibility of the data was within ± 0.5%. The temperature was controlled with a Stelar VTC-91 heater airflow. Additional points in the 0.01 – 10 MHz

frequency range were collected on a Fast-Field Cycling (FFC) Stelar SmarTracer Relaxometer. ^{1}H NMRD profiles were recorded at 283, 298 ad 310 K. All the experiments are repeated three times with a reproducibility of the data within ± 0.5%. The temperature-dependence of the longitudinal relaxivity values was measured at 62 MHz in the 275-320 K range. The concentration of Gd(III) in the solution was determined by using bulk magnetic susceptibility (BMS) shift measurements performed at 11.7 T.[8]

^{1}H NMRD fitting model: For both MB1-2 and KH2-20X, the best fit was achieved by considering a significant contribution from a second-sphere (SS) shell to the relaxivity. Data analysis was conducted by accounting for the presence of two second-sphere water molecules (q^{ss}) positioned at a distance (r^{SS}) of 3.6 Å from the metal ion and in rapid exchange with bulk solvent molecules. To enhance the accuracy of the fit, the rotational dynamics of the SS water molecules within the matrix was described in terms of the Lipari–Szabo model, considering data in the high field region (>3 MHz). Under these conditions, the calculated parameters associated with Gd(III) electron relaxation, Δ^2 and τ_v, can be regarded as fitting parameters without specific physical implications. However, valuable insights can be gained regarding the rotational dynamics. The Lipari-Szabo model considers both the global rotational contribution of the metallo-coiled coil (τ_{RG}) and a faster localized motion (τ_{RL}). The parameter S^2, ranging from zero (independent motions) to one (fully correlated motions), quantifies the degree of correlation between these motions.

Stability test in biological matrix: A stability test for $Gd(MB1\text{-}2)_3$ and Gd(KH2-20X) was carried out *in vitro* under conditions mimicking physiological conditions. For relaxometry studies, 0.45 mg of lyophilized human serum (Seronorm®) was dissolved in 500 µL of the peptide stock solution. Experiments were then carried out on the final solution at pH 7.4 and 298 K.

Circular dichroism spectroscopy: CD spectra were acquired on a Jasco J-1500 Spectropolarimeter using a 1 mm pathlength quartz cuvette for 10 µM peptide trimer solutions, and a 1 cm pathlength quartz cuvette for 1 µM and 0.1 µM peptide trimer solutions, over a wavelength range of 190–300 nm. Prior to experimentation the optical chamber was purged with nitrogen and kept under a nitrogen atmosphere while experiments were running. $TbCl_3$ stock solutions (1 mM, 0.1 mM, and 0.01 mM) were titrated into peptide monomer solutions at corresponding concentrations and buffer conditions: 1 mM $TbCl_3$ into

10 μM peptide in 10 mM HEPES, 0.1 mM $TbCl_3$ into 1 μM peptide in 1 mM HEPES, and 0.01 mM $TbCl_3$ into 0.1 μM peptide in 0.1 mM HEPES (all at pH 7.0). Those experiments at 0.01 mM HEPES concentration had slightly varied pH, ranging from around 7.0 ± 0.3. All solutions were left to equilibrate for, at least, 60 min. (for 10 μM) samples and 6 hours for 1 and 0.1 μM samples, before acquiring the spectra. The observed ellipticity was then converted into molar ellipticity, where the helical content was calculated as the percentage folded, based on the theoretical maximum ellipticity at 222 nm as reported by Scholtz *et al.*[9]

Thermal unfolding data was recorded by monitoring the ellipticity at 222 nm of a 10 μM trimer concentration of MB1-2 and KH2-20X solution in 10 mM HEPES buffer (pH 7.0) in the absence and presence of 10 μM $TbCl_3$, using a Jasco Peltier Type MPTC-513 temperature controller ramping from 20 °C to 85 °C in 0.1°C increments, heating at 1 °C/min. The ellipticity was also monitored upon cooling of the sample back down to 20 °C over the same timeframe.

Folding kinetic experiments were conducted which monitored the ellipticity at 222 nm over 6 hours, for 10 μM, 1 μM and 0.1 μM peptide trimer concentrations after the addition of 1 equiv of $TbCl_3$.

***K_a* determination:** Emission spectra were acquired on a Jasco FP-8500 fluorescence spectrometer using a 1 cm pathlength quartz cuvette. A series of experiments were conducted at different concentrations KH2-20X and MB1-2 trimer (0.1 μM, 1.0 μM, 10 μM) where varying equivalence of $TbCl_3$ were titrated into the solutions. The emission spectra were acquired after the systems reached equilibrium, indicated by stabilization of the Tb(III) signal intensity at 545 nm. The solutions were excited at 280 nm, and the emission spectra was recorded from 455 to 655 nm using a 455 nm long-pass filter. The data was corrected for dilution and the Tb(III) emission peak at 545 nm was integrated and normalized. The normalized data was then plotted as a function of Tb(III) concentration.

Competitive binding studies were carried out as reported by Cotruvo *et al*[10] where the sensitized Tb(III) emission from 3.33 μM KH2-20X was recorded in EGTA buffered Tb(III) solution. The solutions were made up at pH 7.2 (50 mM HEPES buffered) with an ionic strength of 0.1 M (adjusted using KCl) at 20°C. The experiments were ran after 7 days equilibration time where the Tb(III) emission was monitored using the parameters mentioned above. The integrated emission of the Tb(III) at 545 nm for the peptide solution was subtracted from the blank EGTA-buffered solution.[10] The free Tb(III) concentration was

determined as reported periously[10] at the data was fitted to the Hills equation using MatLab.[11]

Stability Studies:

Zinc transmetallation experiments: Emission spectra of Tb(III) were recorded for 1.0 μM solutions of Tb(KH_2-20X) and Tb(MB1-2)$_3$ at 37 °C. Zinc chloride ($ZnCl_2$) was then added to each cuvette to give a final concentration of 125 μM Zn(II), and the fluorescence emission spectra were subsequently collected over time. The Tb(III) emission for each sample was normalized to the integrated intensity of the 545 nm emission peak from the initial spectrum.

Phosphate and HEPES: Emission spectra of Tb(III) were recorded for 1.0 μM solutions of Tb(KH_2-20X) and Tb(MB1-2)$_3$ at 37 °C. Phosphate buffer prepared from a mixture of sodium monobasic and tribasic phosphate salts (to give a stock concentration of 100 mM at pH 7.0) was then added to each cuvette to achieve a final concentration of 1.2 mM phosphate. Fluorescence emission spectra were subsequently collected over time. The Tb(III) emission for each sample was normalized to the integrated intensity of the 545 nm emission peak from the initial spectrum. A control was carried out using the same methodology, this time monitoring the change in Tb emission upon the addition of HEPES buffer instead of phosphate.

Protease experiments: A trypsin (bovine pancreas) stock solution was prepared (1.5 mg/mL) by dissolving in 100 mM HEPES at pH 7.5. The peptide stock solution was prepared by dissolving the lyophilized peptide in the same buffer solution, and the concentration determined as reported previously. In the case when the peptide was metalated, samples were left to equilibrate with the metal upon addition for around 60 min. prior to the addition of the trypsin. Samples were left to incubate in a water bath at 37°C. At specific time points, 35 μl of each sample were taken and quenched with 35 μl of 4% TFA solution. Samples were monitored by analytical HPLC using a linear 0 – 50% MeCN + 0.05% TFA in H2O + 0.05% TFA gradient over 40 min. Individual peaks from the HPLC were collected and then analyzed by ESI mass spectrometry in positive mode. Samples were ionised using nanoelectrospray ionisation (TriVersa NanoMate, Advion Interchim Scientific, Ithaca, NY) installed on a Waters Synapt G2-S Mass Spectrometer (Waters Corporation, Wilmslow, UK), operating at a resolution of 20,000 FWHM.

System preparation for molecular dynamics simulations: A coiled coil structure of three identical MB1-2 peptide sequences was modeled by means of AlphaFold2-multimer v3[12,13] with MMseqs2 available in ColabFold v1.5.5.[14] The Gd(III) ion was placed in a previously characterized metal binding site near Asp and Asn, so that the sidechain oxygens directly coordinate the metal through 9 coordination bonds, schematic shown in S20.[7,15,16]

All titratable residues including Asp, Glu, and Lys were considered fully charged. To mimic the experimental coiled coils, the N- and C-terminus were capped with ACE and NHE capping groups, respectively. The standard protein residues were described with the ff14SB AMBER force field.[17] The non-canonical residues LYL and GLL were parametrized from Lys and Glu, connected with the covalent isopeptide cross-link and capped using a standard acetyl and *N*-methyl capping groups. The restrained electrostatic potential (RESP) fitting procedure was used to derive partial charges from the ESP obtained at the HF/6-31G(d) level of theory. The conformation of the backbone was constrained to the alpha-helix (φ = -60°, ψ = -30.5°) during the geometry optimization at the B3LYP/6-31G(d) level of theory. All quantum-mechanics (QM) calculations were carried out in Gaussian 16 software.[18] A total of four cross-linked coiled coil systems were investigated due to a possibility of the covalent link between Glu and two distinct nearby Lys residues as shown in Figure S5 in the SI.

The TIP3P water model[19] with the corresponding non-bonded 12-6 Lennard-Jones parameters for trivalent Gd(III) ion[20] was used. Each system was solvated in a truncated octahedron box with at least 10 Å of water molecules between the solute and the borders of the box.

Molecular dynamics simulations protocol: The prepared systems were initially minimized for a total of 1000 cycles by first performing 20 cycles of the steepest descent algorithm before switching to the conjugate gradient method for the remaining cycles. The positional restraints with the force constant of 100 kcal mol^{-1} Å^{-2} were applied to the solute during the minimization. The systems were then gradually heated at a constant volume from 100 K to a target temperature of 300 K over 2 ns applying the harmonic positional restraints on the solute atoms with the force constant of 100 kcal mol^{-1} Å^{-2}. The subsequent restrained simulation at a constant pressure was carried out for 2 ns to equilibrate the box density, followed by additional 50 ns NPT simulation without restraints allowing the mutual relaxation of the solute and water. The equilibrated Gd(MB1-2)$_3$ system was subject to 4 repeat unrestrained production MD simulations for 100 ns each, saving the snapshots every 1 ps. All cross-linked Gd(KH2-20X) systems were propagated for 3 repeat production runs for 100

ns each, saving the snapshots every 2 ps. The temperature was maintained at 300 K using Langevin thermostat with a collision frequency of 1 ps^{-1} and the pressure was controlled using Monte Carlo barostat with isotropic position scaling. The SHAKE algorithm was used to constrain bonds involving hydrogen atoms.[21] The non-bonded cutoff of 10 Å was used in combination with Particle Mesh Ewald (PME) method to account for long-range electrostatic interactions. The 2 fs time step was used in all simulations. All MD simulations and analysis were carried out using the *pmemd.cuda* and the *cpptraj* modules of the AMBER 22 software, respectively.[18] The C-alpha root mean square deviation (RMSD) analysis was carried out to gain insights into conformational stability and dynamics of the coiled coils with respect to the reference AlphaFold2 structure. Principal component analysis (PCA) was carried out using the cartesian coordinates of the backbone atoms (N, C-alpha, C, O) extracted from MD trajectories of all systems. Free energy landscape (FEL) analysis tool was used to generate FEL along the PC1 and PC2.[22] Radial distribution function (RDF) of water hydrogens from Gd(III) was calculated to investigate the hydration of the Gd(III) metal site. Final RDFs were obtained by averaging the RDFs calculated for each of the two hydrogen atoms ($H1_{Wat}$ and $H2_{Wat}$) from water molecules separately. To assess the persistence of specific water molecules interacting within the second coordination sphere of Gd(III) during the MD simulations, we tracked the distance between the oxygen atom of each water molecule (O_{Wat}) and the Gd(III) ion over the entire simulation trajectory of $Gd(MB1\text{-}2)_3$ and Gd(KH2-20X) A_3. Interactions were defined based on a cutoff distance, and the duration for which individual water molecules remained continuously within this cutoff was calculated to determine their interaction lifetimes. All structures were visualized using PyMOL v2.5.4.[23]

Scheme S1: Reaction scheme of KH2-20X

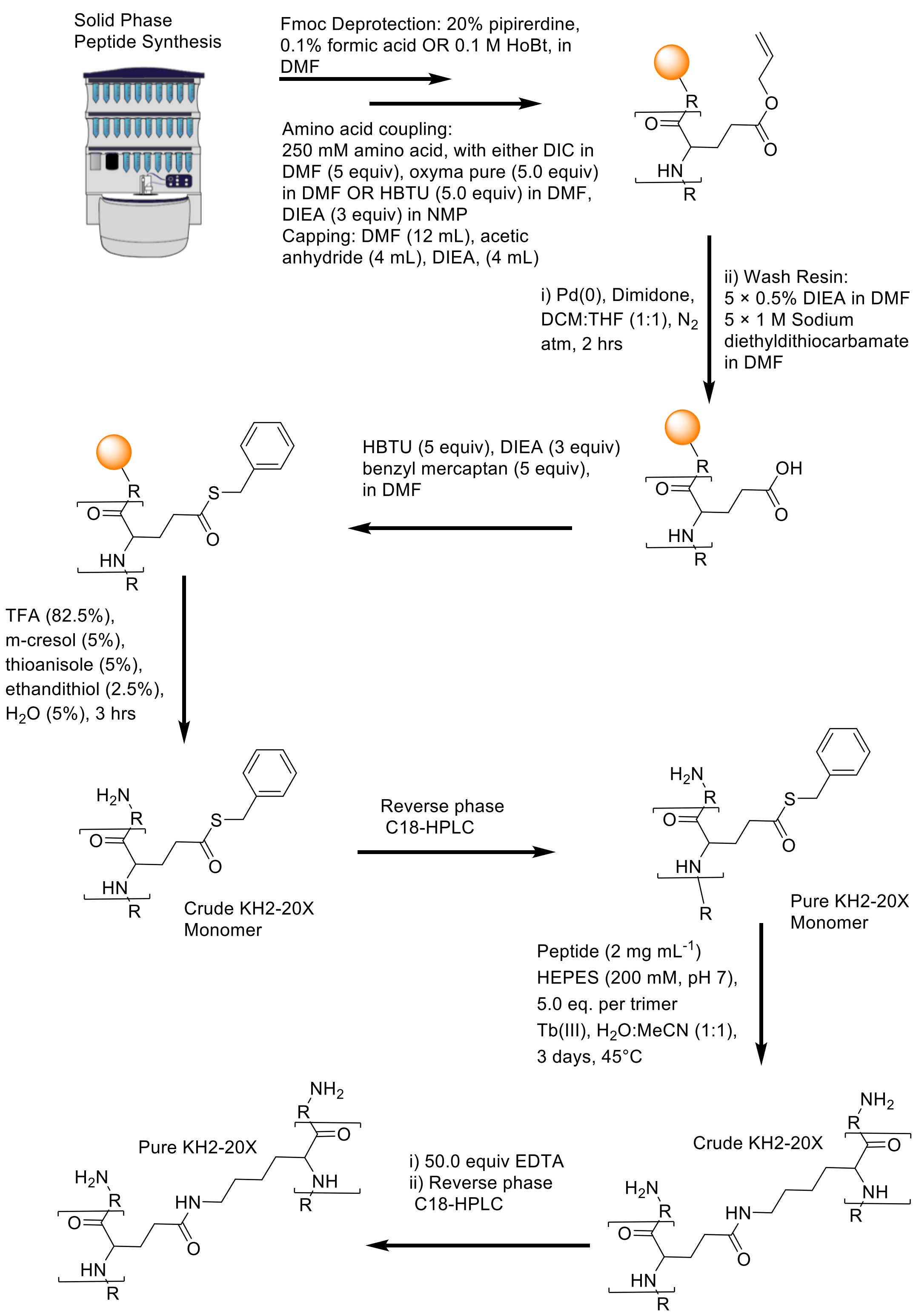

Scheme S1: Shows the synthesis of the KH2-20X monomer and the KH2-20X trimer, in the presence of a Tb(III) template. Shown in orange is the solid phase support (resin) located at the C-terminus.

Figure S1: HPLC and MS monitoring of KH2-20X cross-linking reaction

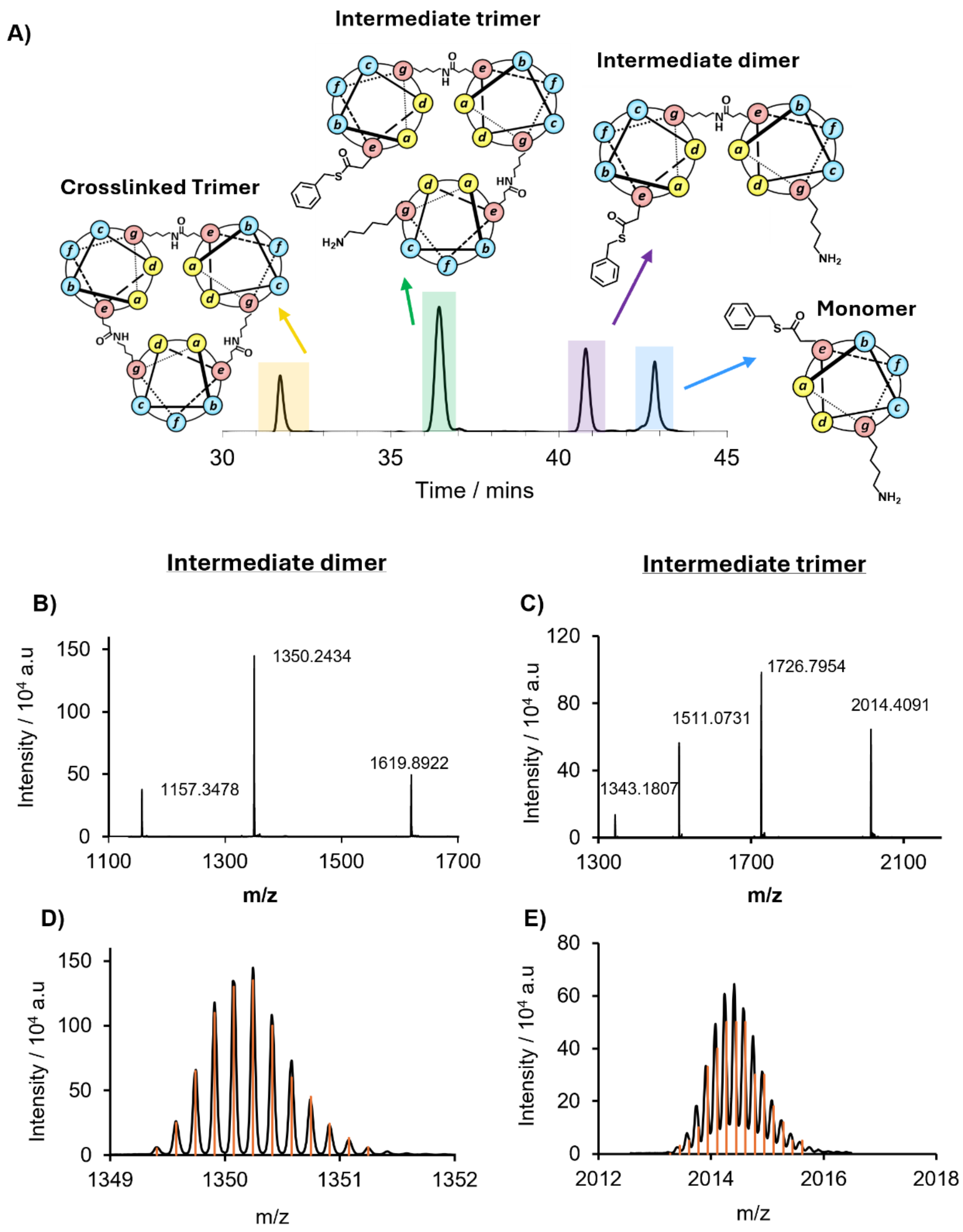

Figure S1: A) Analytical C18-reverse phase HPLC trace of the cross-linking reaction mixture after 3 days at r.t., showing peaks corresponding to the monomer, intermediate dimer and trimer species, and fully cross-linked trimer. A linear gradient of 0–60% MeCN + 0.05% TFA in H_2O + 0.05% TFA was applied over 60 min., and absorbance was monitored at 210 nm. Electrospray ionization mass spectra for B) the intermediate cross-linked dimer and C) the intermediate cross-linked trimer. The $[M+6H]^{6+}$ experimental (black) and theoretical (orange) isotopic distributions for the intermediate D) dimer and E) trimer, respectively. Characterization data for the monomer and fully cross-linked trimer are shown in Figure S2.

Figure S2: Analytical HPLC and MS characterization of KH2-20X monomer and trimer

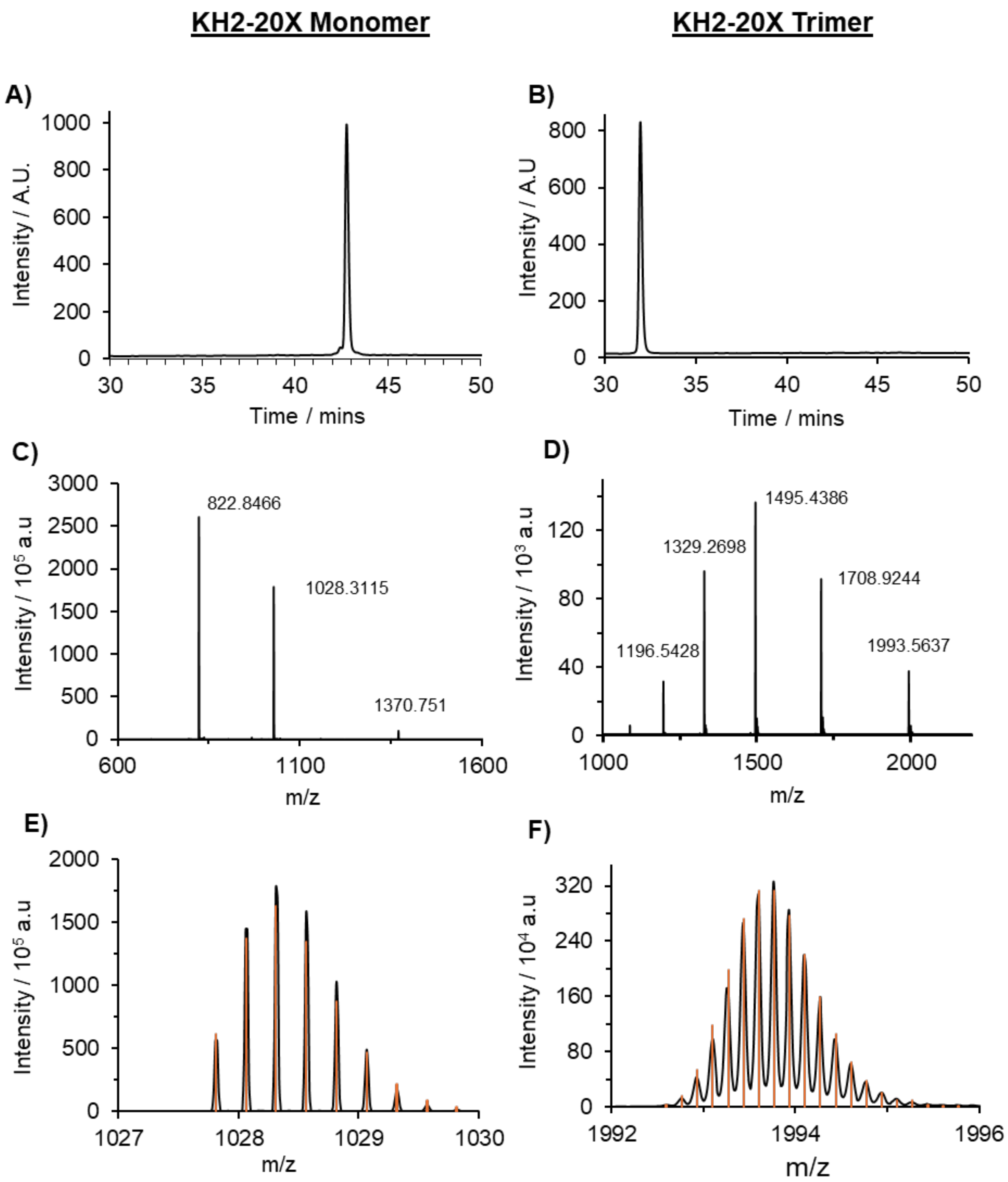

Figure S2: Analytical C18-reverse phase HPLC trace (top) of purified KH2-20X thioester monomer (left) and KH2-20X cross-linked trimer (right) using a linear 0 – 60% MeCN + 0.05% TFA in H_2O + 0.05% TFA gradient over 60 min. and monitored at 210 nm. Electrospray ionization mass spectra (middle), and the experimental (black) and theoretical (orange) isotopic distributions (bottom) are shown for the KH2-20X thioester monomer $[M+4H]^{4+}$ charge peak (left), and the KH2-20X trimer $[M+6H]^{6+}$ charge peak (right). The average mass of the KH2-20X thioester monomer and fully cross-linked trimer are 4109.8 and 11956.6 Da, respectively.

Figure S3: Native mass spectrometry of Tb/Gd(III) metalated MB1-2 and KH2-20X

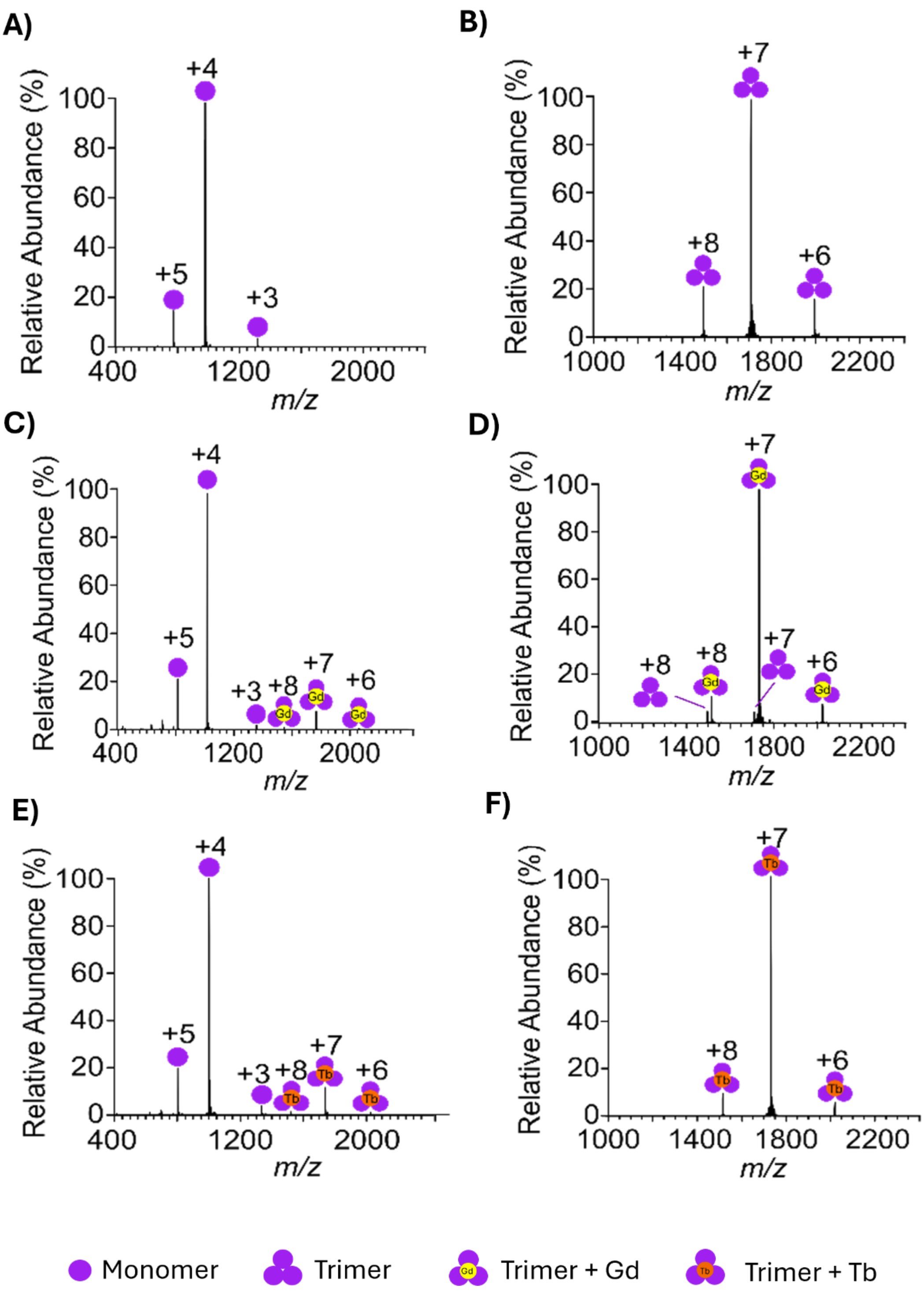

Figure S3: Native mass spectrometry analysis of 10 µM MB1-2 and KH2-20X with Gd(III) and Tb(III) in 50 mM ammonium acetate at pH 6.8. Mass spectra of monomeric MB1-2 peptide (purple circle), KH2-20X peptide trimer in the absence (A,B) and presence equimolar amounts of Gd(III) (yellow circle) (C,D) or Tb(III) (orange circle) (E,F).

Table S1: The average theoretical and observed masses of MB1-2 and KH2-20X, determined using native MS, in both unbound and metal-bound complexes with Tb(III) and Gd(III). The error on the mass calculated between charge states in all cases was ≤ 0.03 Da.

Complex	Theoretical Mass (Da)	Observed Mass (Da)
MB1-2 monomer	4003.6	4003.2
MB1-2 trimer + Gd	12167.9	12167.5
MB1-2 trimer + Tb	12169.6	12169.6
KH2-20X	11956.6	11956.6
KH2-20X + Gd	12113.8	12114.2
KH2-20X + Tb	12115.5	12115.5

Figure S4: Protocol for molecular dynamics studies: Non-cross-linked Gd(MB1-2)$_3$

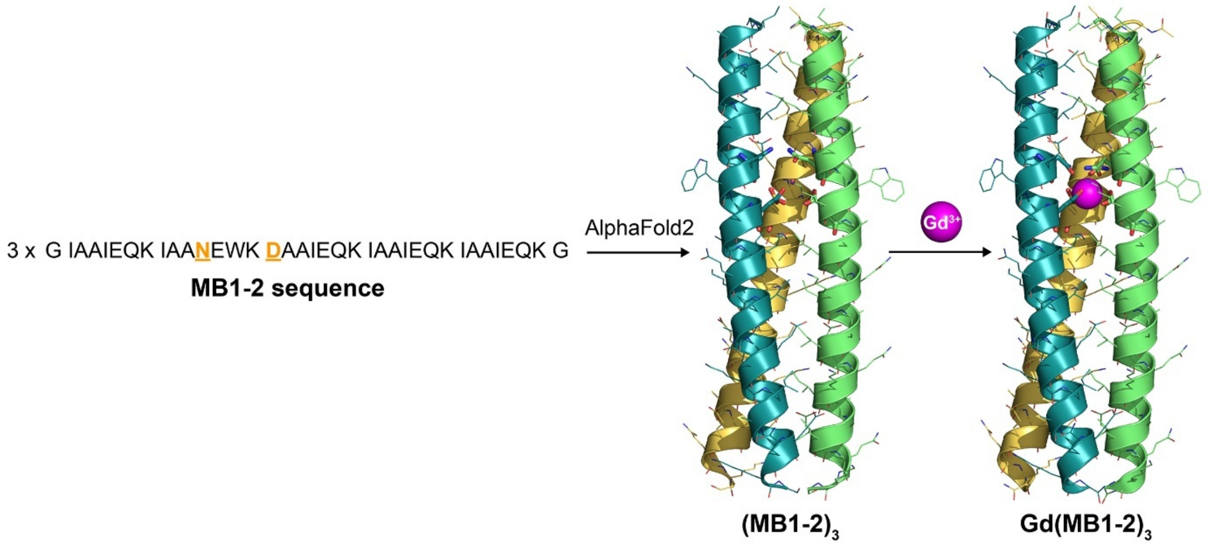

Figure S4: Protocol for generating a coiled coil structure of Gd(MB1-2)$_3$. Asparagine and aspartate residues that coordinate metal ion are highlighted orange in the sequence. The Gd(III) is shown as a magenta sphere.

Figure S5: Protocol for molecular dynamics studies: Cross-linked Gd(KH2-20X)

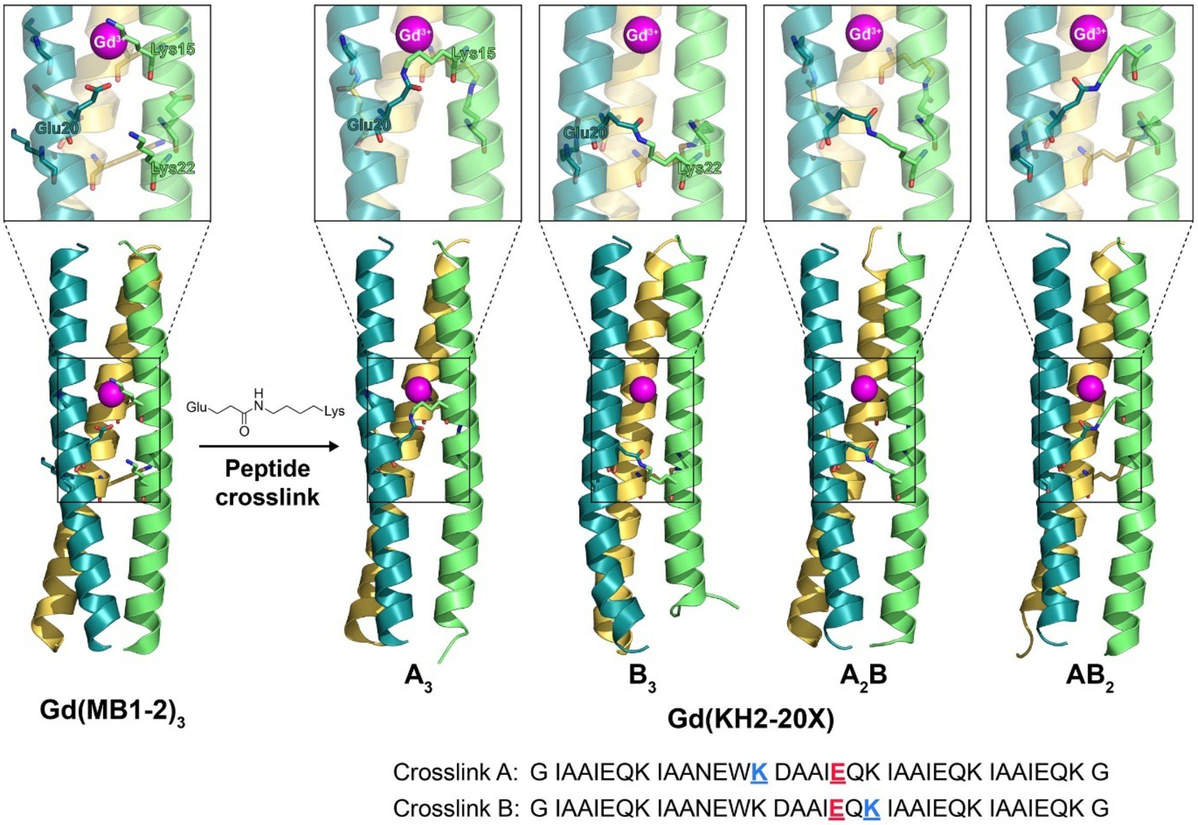

Figure S5: Protocol for constructing the cross-linked Gd(KH2-20X) structure. Each chemically labeled glutamate residue (Glu_{20}) can form an isopeptide bond with a proximal lysine residue at position A (Lys_{15}) or B (Lys_{22}), resulting in four possible cross-linked configurations: A_3, B_3, A_2B and AB_2. The Glu and Lys residues involved in the cross-link are highlighted red and blue in the sequence, respectively. The Gd(III) is shown as a magenta sphere.

Figure S6: RMSD calculations for Gd(MB1-2)$_3$ and Gd(KH2-20X)

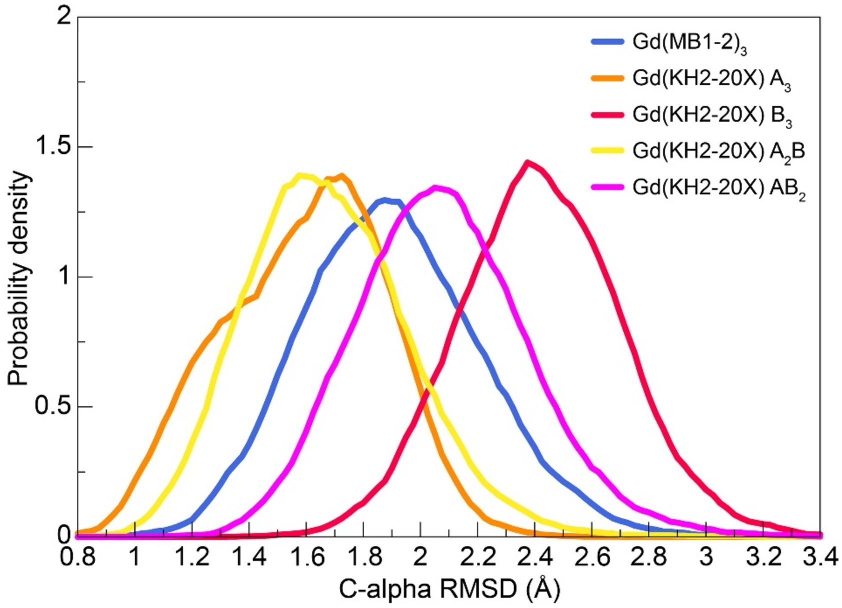

Figure S6: Normalized histograms of the backbone C-alpha root mean square deviation (RMSD) calculated from MD simulations of Gd(MB1-2)$_3$ and Gd(KH2-20X) systems with respect to the reference AlphaFold2 structures generated according to Figures S4 and S5.

Figure S7: Free energy landscape calculations for Gd(MB1-2)$_3$ and Gd(KH2-20X)

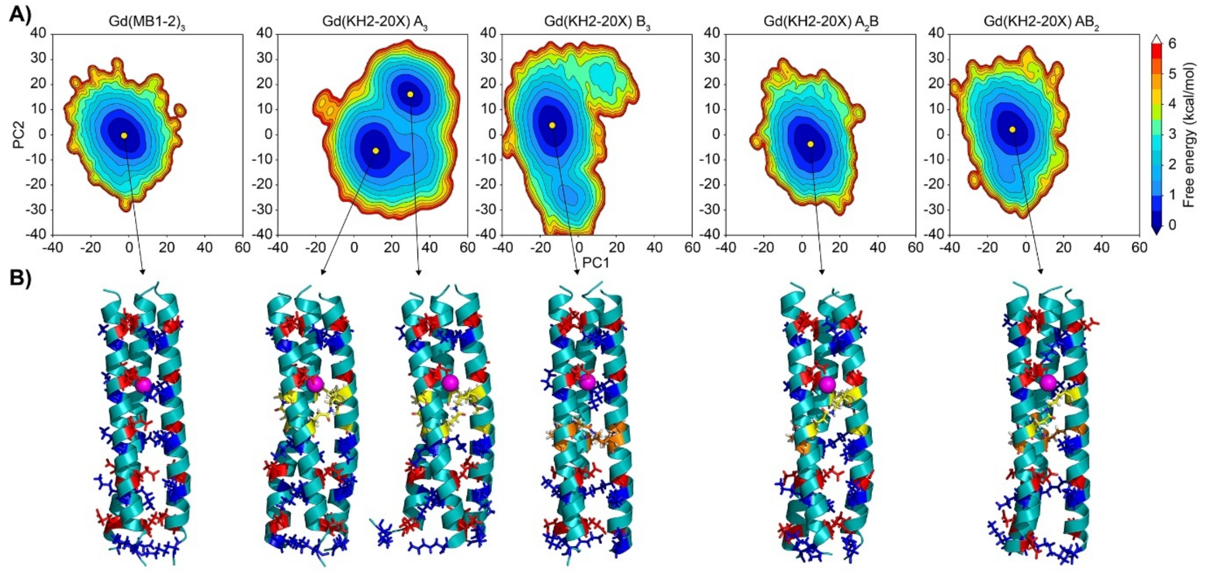

Figure S7: A) Free energy landscape (FEL) at 300 K projected on first two principal components PC1 and PC2 calculated from MD simulations of Gd(MB1-2)$_3$ and Gd(KH2-20X) systems. B) Representative conformations of Gd(MB1-2)$_3$ and Gd(KH2-20X) systems. Positively charged Lys and negatively charged Glu are shown in blue and red, respectively. Cross-link type A and B are shown as yellow and orange, respectively. The Gd(III) is shown as a magenta sphere.

Figure S8: Peptide sequencing by trypsin degradation

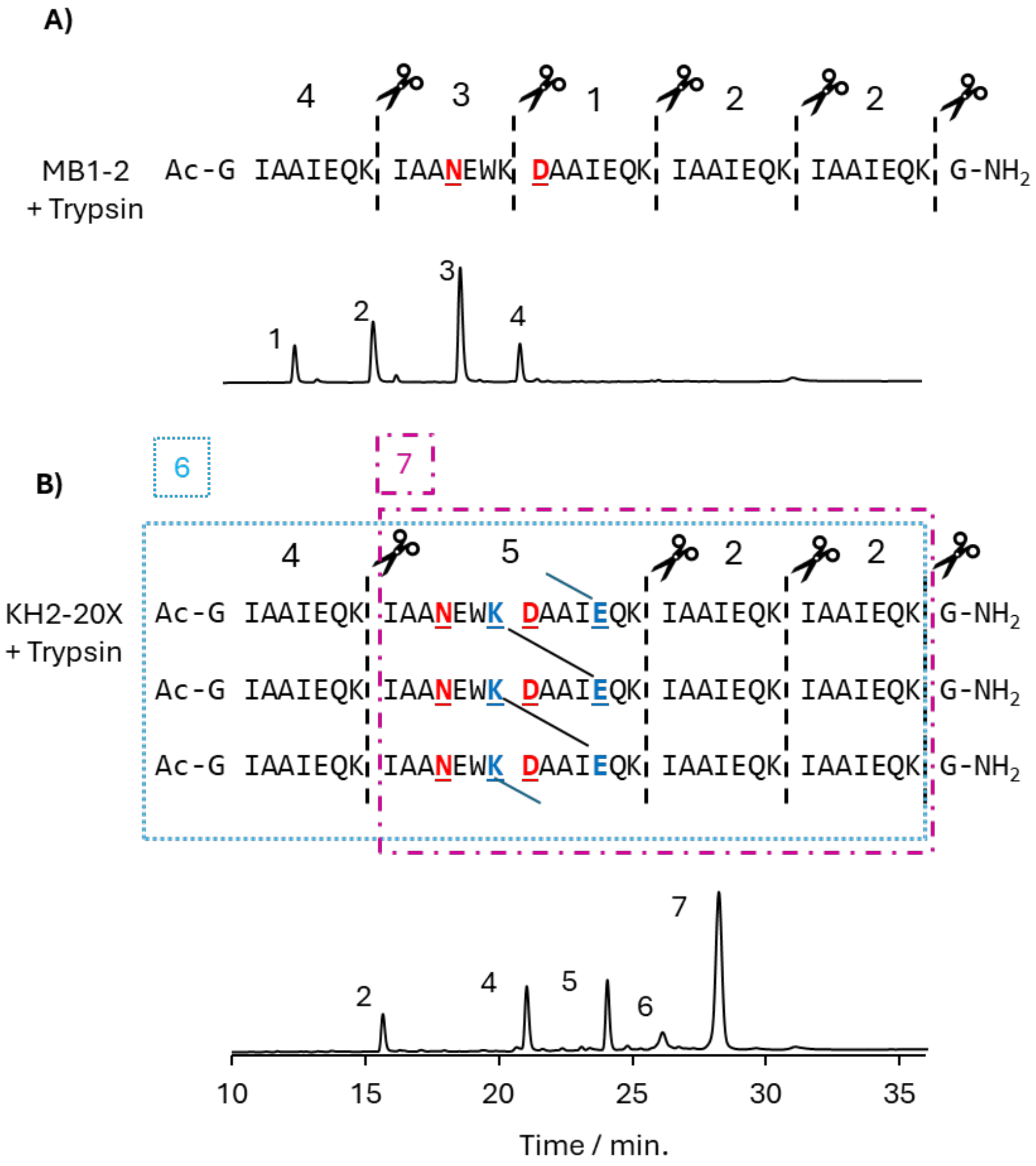

Figure S8: Analytical C18-reverse phase HPLC spectra of 100 µM trimer solutions of A) MB1-2 and B) KH2-20X following incubation with 12 µM trypsin in 100 mM HEPES buffer pH 7.5 over 3 hours. Proteolytic digestion was quenched by the addition of 4% (v/v) TFA. A linear gradient of 0 to 50% MeCN + 0.05 % TFA was applied over 40 min. Individual peaks were collected and analyzed by ESI mass spectrometry to assign peptide fragments (see Figure S9). Black, blue, and pink dashed lines indicate specific sites of proteolytic cleavage, with the numbered labels corresponding to the respective fragment peaks observed in the HPLC traces.

Figure S9: Mass Spectra of peptide fragments from MB1-2 and KH2-20X trypsin degradation

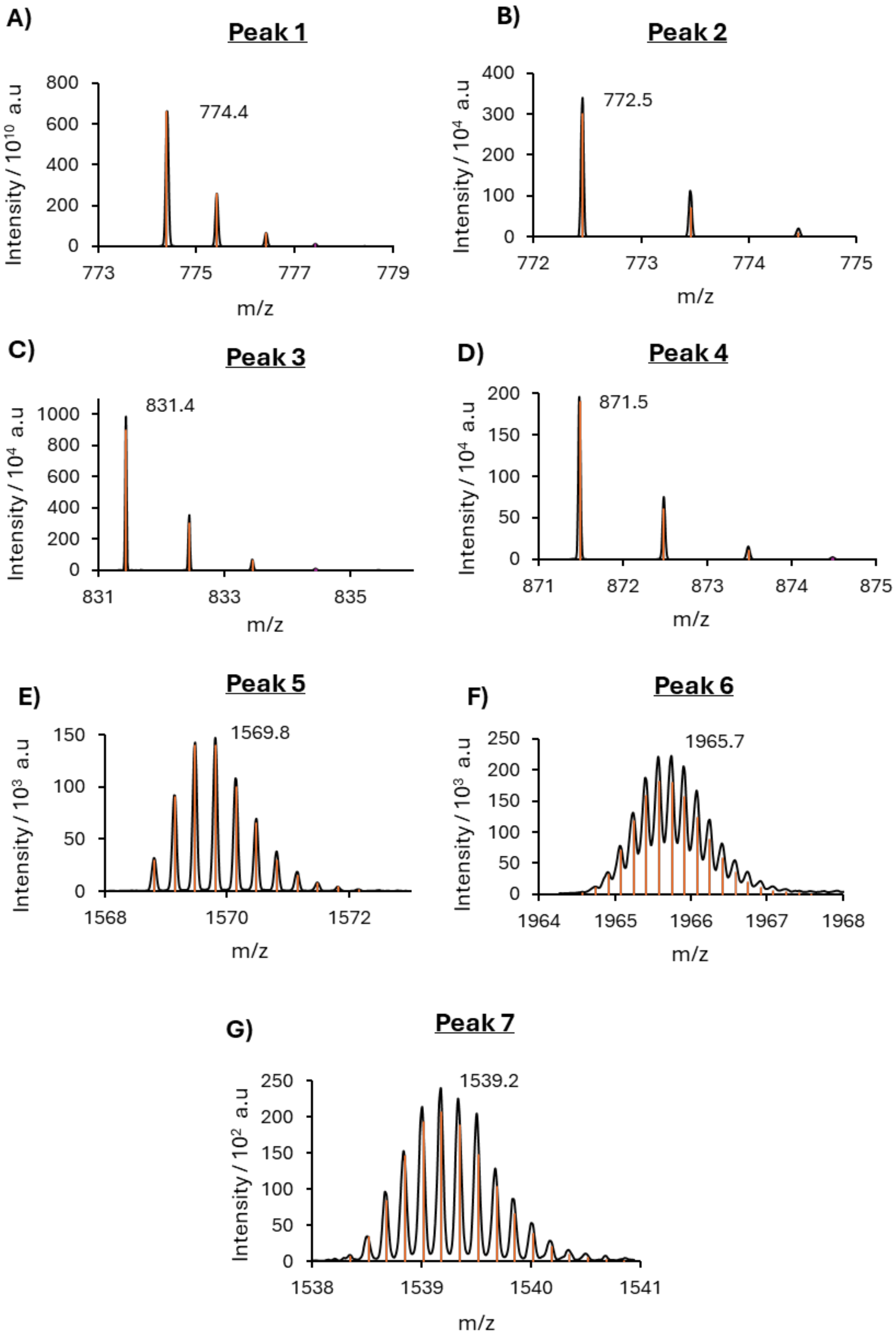

Figure S9: Electrospray ionization mass spectra (black) and theoretical (orange) isotopic distribution for the corresponding HPLC peaks from Figure S8 and Table S2, as follows: A) HPLC peak 1, $[M+1H]^{1+}$ B) peak 2, $[M+1H]^{1+}$ C) peak 3, $[M+1H]^{1+}$ D) peak 4, $[M+1H]^{1+}$ E) peak 5, $[M+3H]^{3+}$ F) peak 6, $[M+6H]^{6+}$ G) peak 7, $[M+6H]^{6+}$.

Table S2: The theoretical and observed mass of peptide fragments from the degradation of KH2-20X and MB1-2 in trypsin and their corresponding sequences

Peak	Retention Time / mins[a]	Excepted Average Mass $[M+H]^+$/ Da	Observed Average Mass $[M+H]^+$/ Da	Fragment Structure
1	12.3	774.4	774.4	**D**AAIEQK
2	15.1	772.5	772.5	IAAIEQK
3	18.5	831.4	831.4	IAA**N**EWK
4	20.8	871.5	871.5	Ac-G IAAIEQK
5	23.6	4706.2	4706.4	IAA**N**EWK **D**AAIEQK<br>IAA**N**EWK **D**AAIEQK<br>IAA**N**EWK **D**AAIEQK
6	26.3	11787.4	11787.4	Ac-G IAAIEQK IAA**N**EWK **D**AAIEQK IAAIEQK IAAIEQK<br>Ac-G IAAIEQK IAA**N**EWK **D**AAIEQK IAAIEQK IAAIEQK<br>Ac-G IAAIEQK IAA**N**EWK **D**AAIEQK IAAIEQK IAAIEQK
7	28.3	9229.1	9225.1	IAA**N**EWK **D**AAIEQK IAAIEQK IAAIEQK<br>IAA**N**EWK **D**AAIEQK IAAIEQK IAAIEQK<br>IAA**N**EWK **D**AAIEQK IAAIEQK IAAIEQK

[a] Based on C18-analytical RP-HPLC with a linear gradient of 0 to 50% MeCN + 0.05% TFA in H_2O to 0.05% TFA gradient over 40 min., monitored at 210 and 280 nm.

Figure S10: Circular dichroism folding data

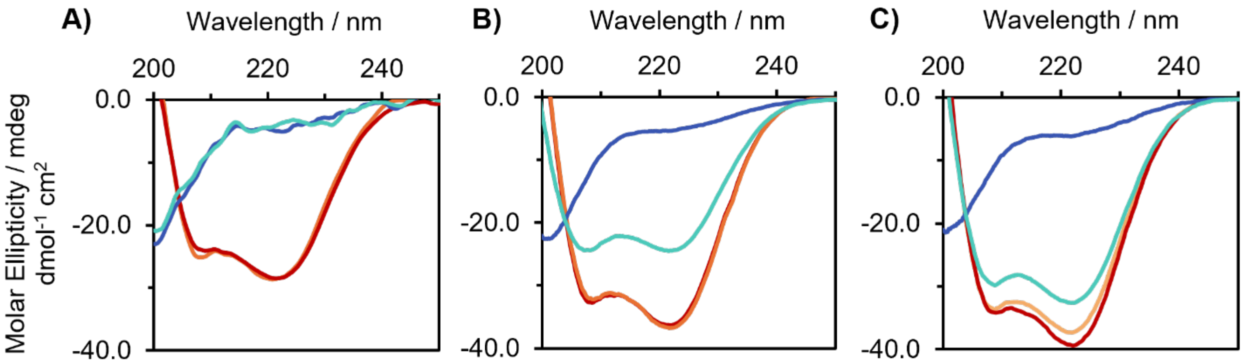

Figure S10: CD spectra of KH2-20X and MB1-2 trimers at varying concentrations: A) 0.1 µM, B) 1.0 µM, and C) 10.0 µM. Spectra are shown for apo KH2-20X (orange), KH2-20X with 1.0 eq $TbCl_3$ (red), apo MB1-2 (blue), and MB1-2 with 1.0 eq $TbCl_3$ (turquoise). Experiments were conducted at 293 K in HEPES buffer at pH 7.0, with buffer concentrations adjusted to match peptide concentration (0.1 mM, 1.0 mM, and 10.0 mM for 0.1 µM, 1.0 µM, and 10.0 µM peptide, respectively). The 1.0 µM samples experienced a slight pH drift to 7.3 due to the lower buffer capacity. Similarly, for the 0.1 µM samples, pH drifted between 6.8 and 7.3 by the end of the measurement. All spectra were recorded in triplicate and smoothed using a Gaussian function.

Figure S11: CD kinetic data MB1-2 and KH2-20X

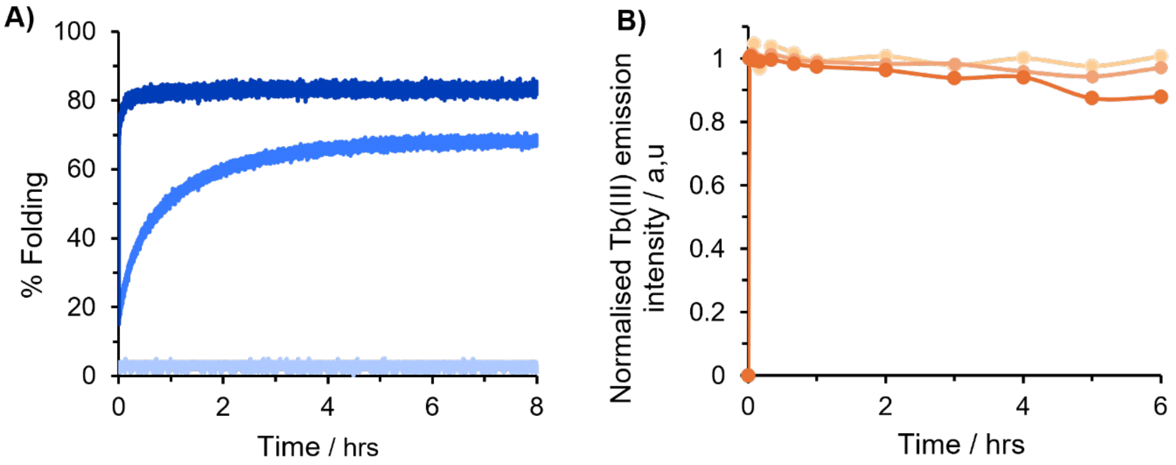

Figure S11: A) Folding, determined from the 222 nm CD signal, monitored as a function of time on addition of 1.0 equiv of $TbCl_3$ to 10 µM MB1-2 trimer in 10 mM HEPES (dark blue), 1.0 µM MB1-2 trimer in 1.0 mM HEPES (blue), and 0.1 µM MB1-2 trimer in 0.1 mM HEPES (light blue). All samples were prepared at pH 7.0, however the pH of the 0.1 µM sample was seen to have drifted to 7.3 after 8 hours. B) The normalized emission intensity from the integrated Tb(III) emission peak at 545 nm as a function of time on addition of 1.0 equiv of $TbCl_3$ to 10 µM (dark orange), 1.0 µM (orange) and 0.1 µM (light orange) KH2-20X, in 10 mM HEPES buffer pH 7.0.

Figure S12: Fluorescence Tb(III) binding titrations of MB1-2 and KH2-20X

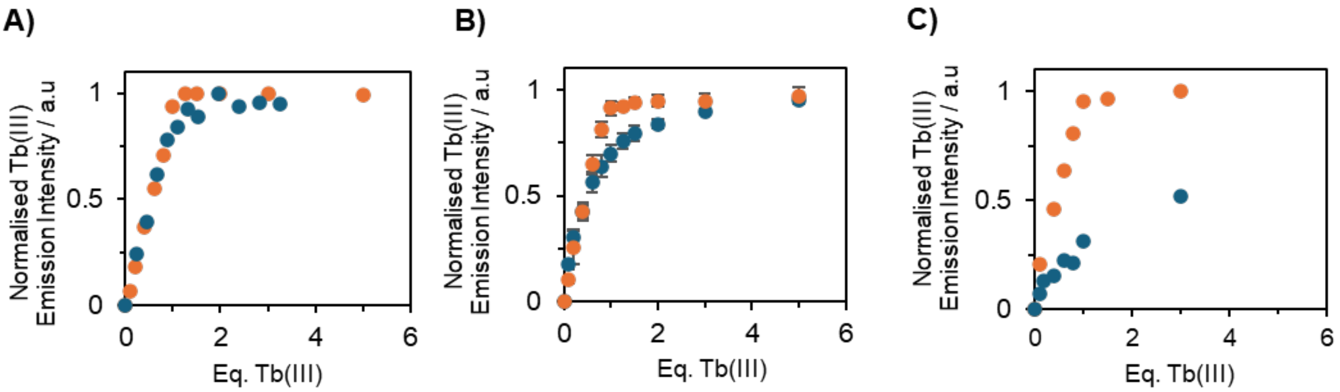

Figure S12: Tb(III) binding titrations comparing MB1-2 and KH2-20X. Normalized integrated emission intensity at 545 nm is plotted as a function of Tb(III) equivalents per trimer for KH2-20X (orange) and $(MB1\text{-}2)_3$ (blue) at (A) 10.0 μM, (B) 1.0 μM, and (C) 0.1 μM trimer concentration. Measurements were conducted in 10 mM HEPES buffer pH 7.0, at 293 K, and with λ_{exc} = 280 nm.

Figure S13: Phosphate and Zn(II) kinetic stability studies

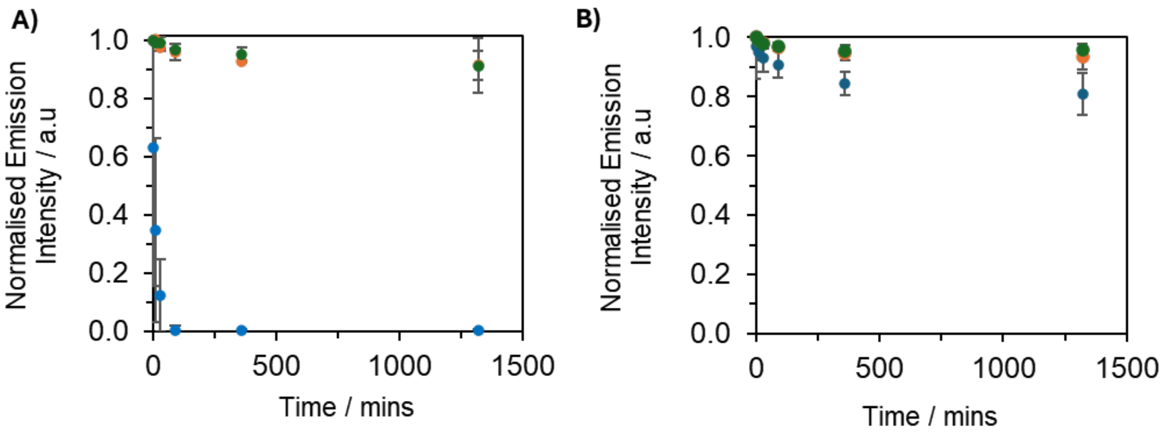

Figure S13: Shows the normalized emission intensity as a function of time for 1 μM A) Tb(MB1-2)$_3$ and B) Tb(KH2-20X) after the addition of 1.2 mM HEPES pH 7.0 (orange, control), 1.2 mM phosphate pH 7.0 (blue) or 125 μM $ZnCl_2$ in 50 mM HEPES pH 7.0 (green). All experiments were carried out at 310 K. Error bars determined from standard deviation error of three independent repeat experiments.

Figure S14: MB1-2 degradation studies with trypsin

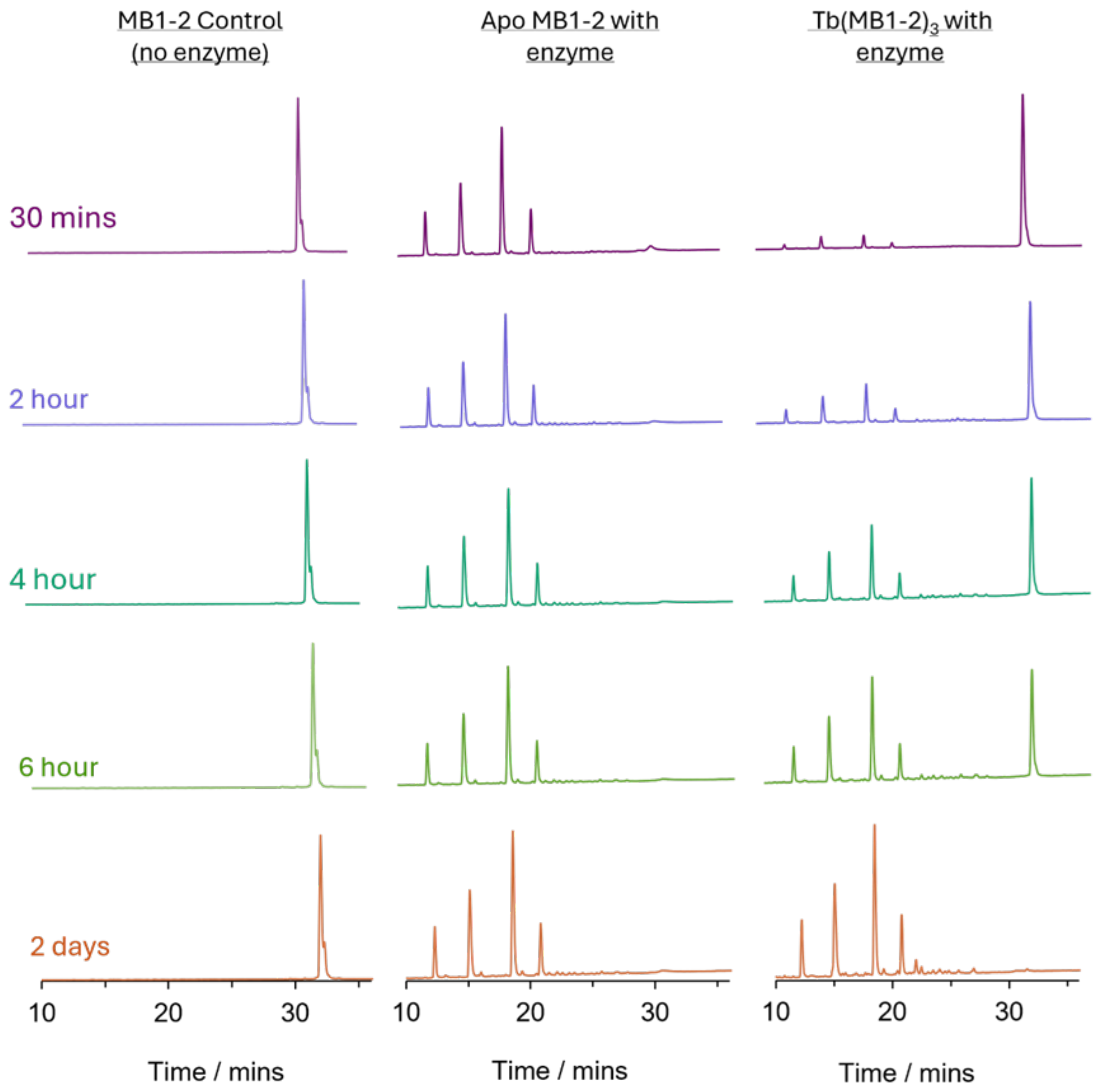

Figure S14: Analytical C18-reverse phase HPLC trace of 100 μM MB1-2 trimer control (no enzyme) and apo and metalated MB1-2 trimer in the presence of 12 μM trypsin (bovine pancreas) and 100 mM HEPES buffered at pH 7.5. A linear 0 – 50% MeCN + 0.05% TFA in H_2O + 0.05% TFA gradient over 40 min. was used where the 210 nm wavelength was monitored.

Figure S15: KH2-20X degradation studies with trypsin

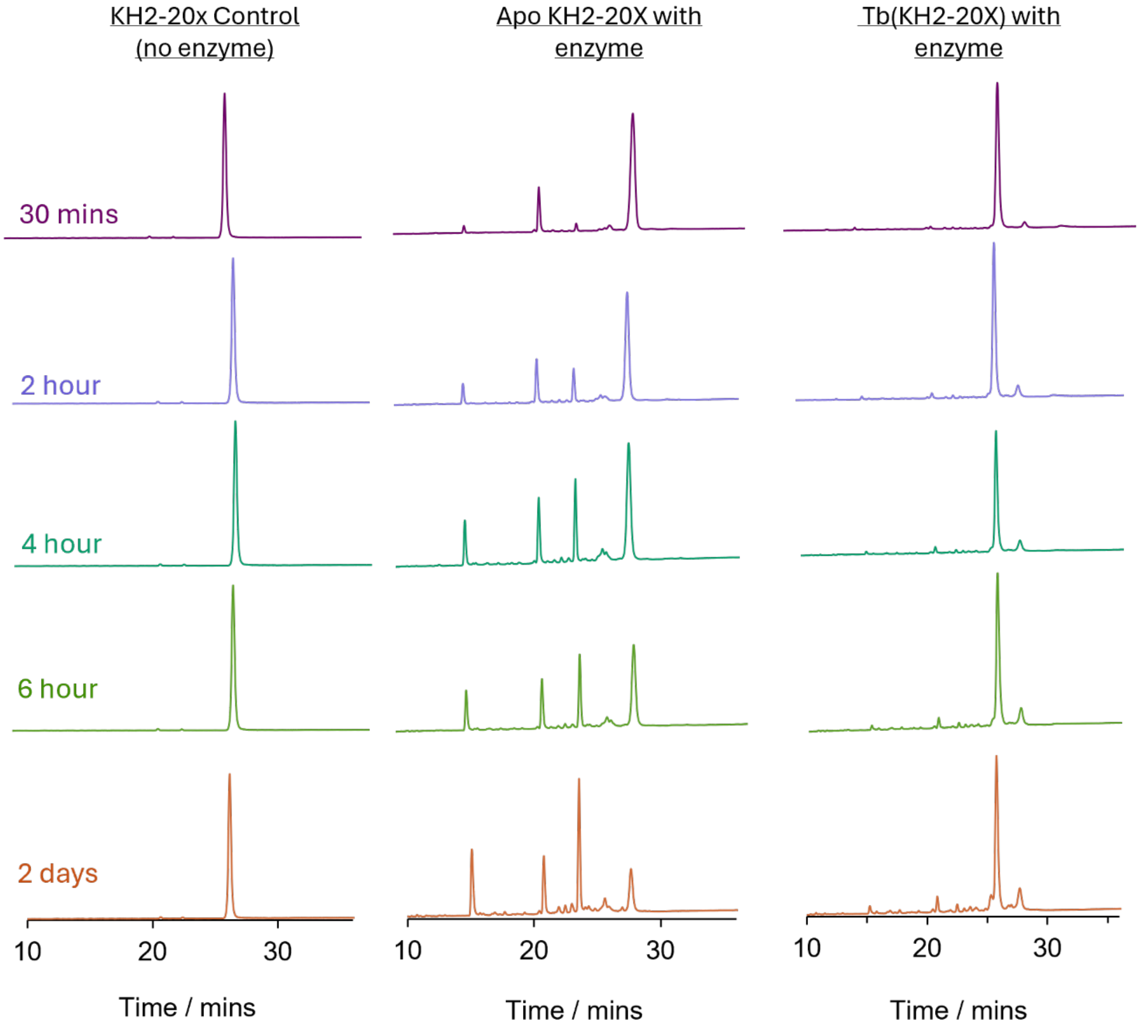

Figure S15: Analytical C18-reverse phase HPLC trace of 100 μM KH2-20X control (no enzyme) and apo and metalated KH2-20X in the presence of 12 μM trypsin (bovine pancreas) and 100 mM HEPES buffered at pH 7.5. A linear 0 – 50% MeCN + 0.05% TFA in H_2O + 0.05% TFA gradient over 40 min. was used where the 210 nm wavelength was monitored.

Figure S16: Relaxivity data for Gd(KH2-20X) and Gd(MB1-2)$_3$

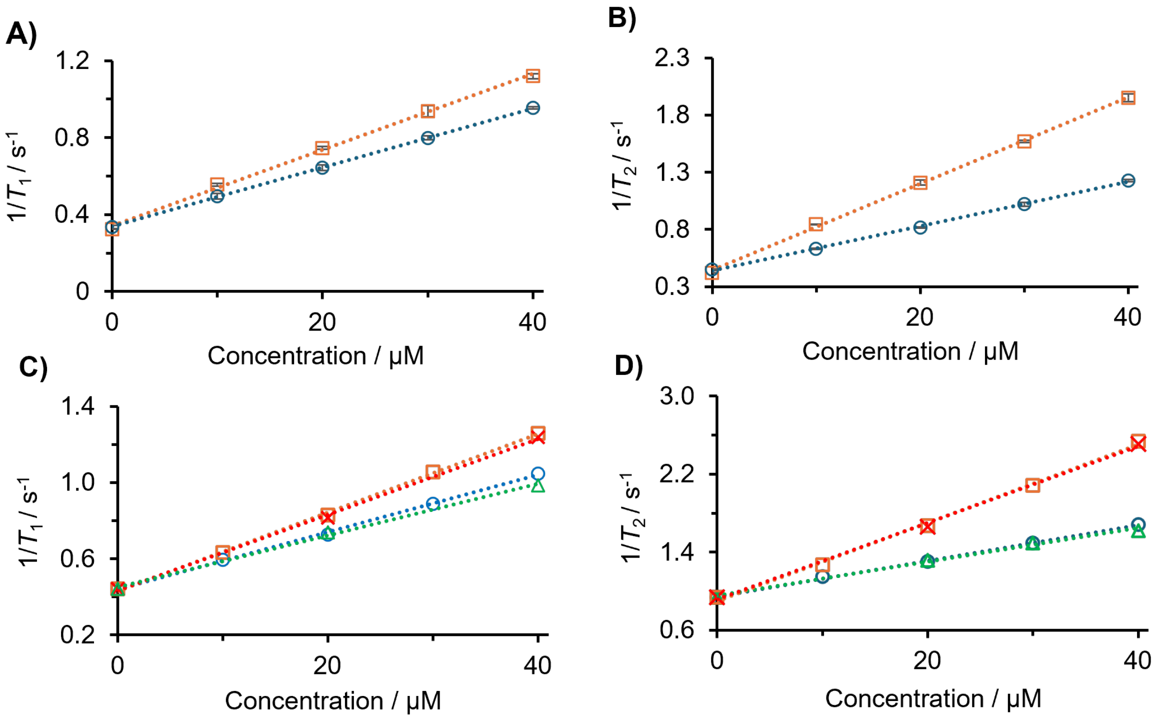

Figure S16: 60 MHz ^{1}H magnetic resonance relaxivity plots showing the reciprocal of T_1 (A, C) and T_2 (B, D) as a function of concentration for Gd(MB1-2)$_3$ (blue) and Gd(KH2-20X) (orange). Panels A and B represent data in the absence of 0.6 mM lyophilized human serum, while panels C and D show data in its presence following 2 hours of incubation (Gd(MB1-2)$_3$, blue; Gd(KH2-20X), orange) and after 3 days of incubation (Gd(MB1-2)$_3$, green; Gd(KH2-20X), red). Gd(MB1-2)$_3$ and Gd(KH2-20X) samples prepared for Gd(III) in the presence of 5 equivalents peptide trimer. Samples recorded at 293 K, in the presence of 100 mM HEPES buffer pH 7.0 and on a 60 MHz NMR spectrometer. Error bars determined from standard deviation error of three independent repeat experiments.

Figure S17: ^{1}H NMRD profiles of Gd(MB1-2)$_3$ and Gd(KH2-20X)

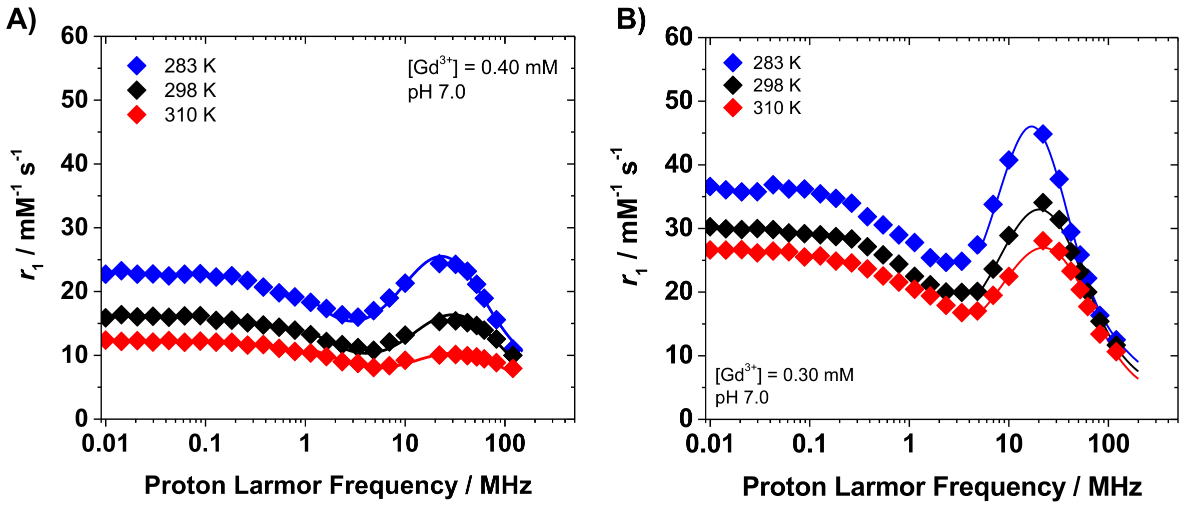

Figure S17: ^{1}H NMRD profiles of A) Gd(MB1-2)$_3$ and B) Gd(KH2-20X) at different temperatures. Solid lines are calculated from the parameters from Table S3. Experiments were conducted in 300 mM HEPES buffered at pH 7.0 with a Gd(III) to peptide trimer ratio of 1:5 at a Gd(III) concentration of around 0.4 mM for MB1-2 and 0.3 mM for KH2-20X.

Figure S18: 62 MHz r_1 relaxivity temperature dependence studies

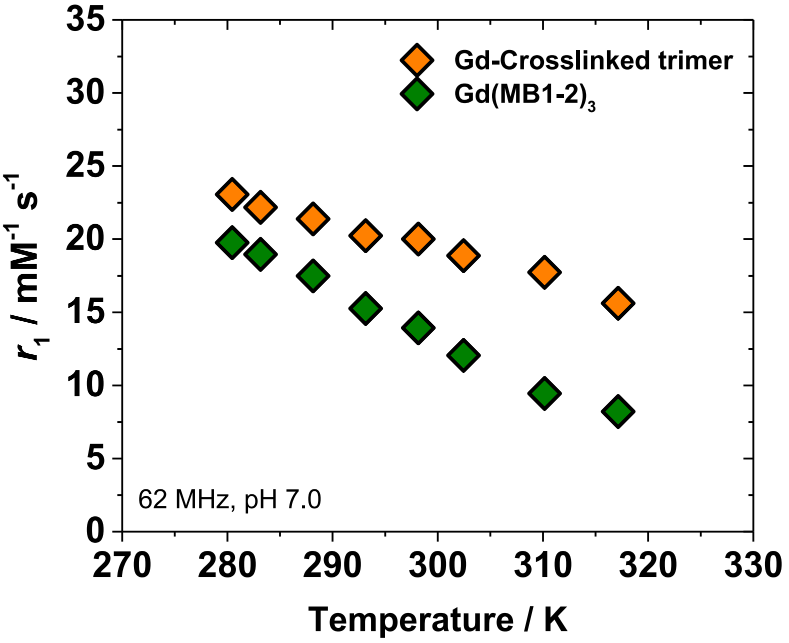

Figure S18: Temperature dependence of r_1 measured for Gd(MB1-2)$_3$ (green) and the Gd(KH2-20X) (orange) at 62 MHz providing insight into the second-sphere water exchange process. The observed decrease in r_1 with increasing temperature indicates a fast water exchange regime. Experiments were conducted in 300 mM HEPES buffered at pH 7.0 with a Gd(III) to peptide trimer ratio of 1:5 at a Gd(III) concentration of around 0.4 mM for MB1-2 and 0.3 mM for KH2-20X.

Figure S19: ^{1}H NMRD r_2 relaxivity studies

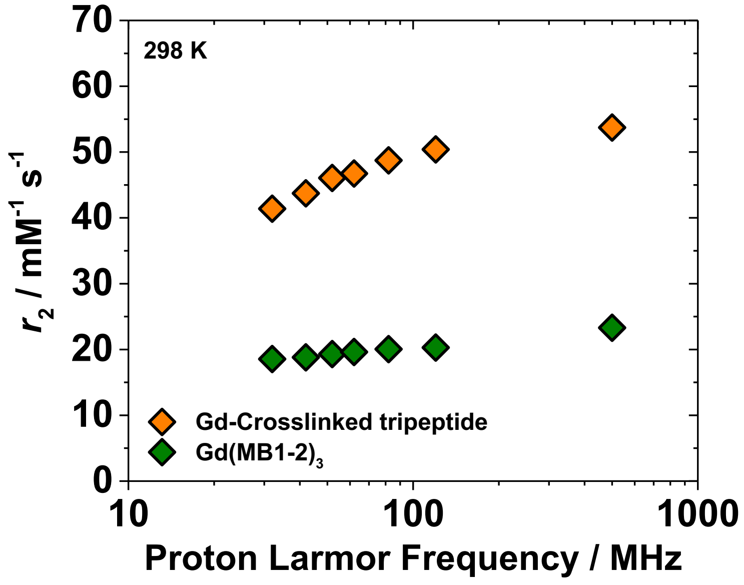

Figure S19: r_2 values for Gd(MB1-2)$_3$ and the Gd(KH2-20X) measured in the 30-500 MHz range at 298 K. Experiments were conducted in 300 mM HEPES buffered at pH 7.0 with a Gd(III) to peptide trimer ratio of 1:5 at a Gd(III) concentration of around 0.4 mM for MB1-2 and 0.3 mM for KH2-20X.

Table S3: Best fit parameters from the analysis of ^{1}H NMRD data

Parameters	$Gd(MB1\text{-}2)_3$	Gd(KH2-20X)
$^{20}r_1$ / $mM^{-1}s^{-1}$	15.3	34.1
τ_{RL} / ps	184 ± 10	318 ± 37
τ_{RG} / ns	5[a]	7[a]
S^2	0.30 ± 0.02	0.45 ± 0.01
τ_M / ns	3.0 ± 0.1	5.0 ± 0.1
q^{ss}	2.0[a]	2.0[a]
r^{ss} / Å	3.6[a]	3.6[a]
a / Å	4[a]	4[a]
^{298}D / 10^{-10} m^2 s^{-1}	2.3[a]	2.3[a]

[a] fixed during the analysis.

$^{20}r_1$ - Longitudinal relaxation rate (at 20 Hz); τ_{RL} - Local rotational correlation time; τ_{RG} - Global rotational correlation time; S^2 - Order parameter: degree of correlation between τ_{RL} and τ_{RG}; τ_M^{SS} – Second-sphere residency time; q^{ss} – Second-sphere Gd(III) hydration state; r^{ss} – Second-sphere water to Gd(III) bond distance; a - The distance of closest approach for outer-sphere water molecules; ^{298}D - Relative diffusion coefficient.

Figure S20 Radial distribution function of water in the SS for Gd(MB1-2)$_3$ and Gd(KH2-20X)

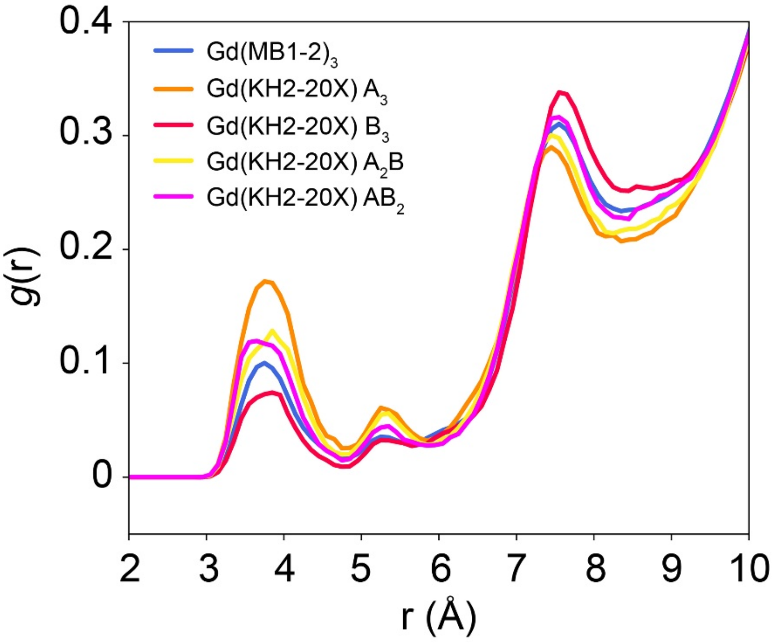

Figure S20: Radial distribution function (RDF) of water hydrogen atoms (H_{Wat}) from Gd(III) in Gd(MB1-2)$_3$ and Gd(KH2-20X) systems.

Figure S21: Radial Distribution Functions of water hydrogen atoms from Gd(III)

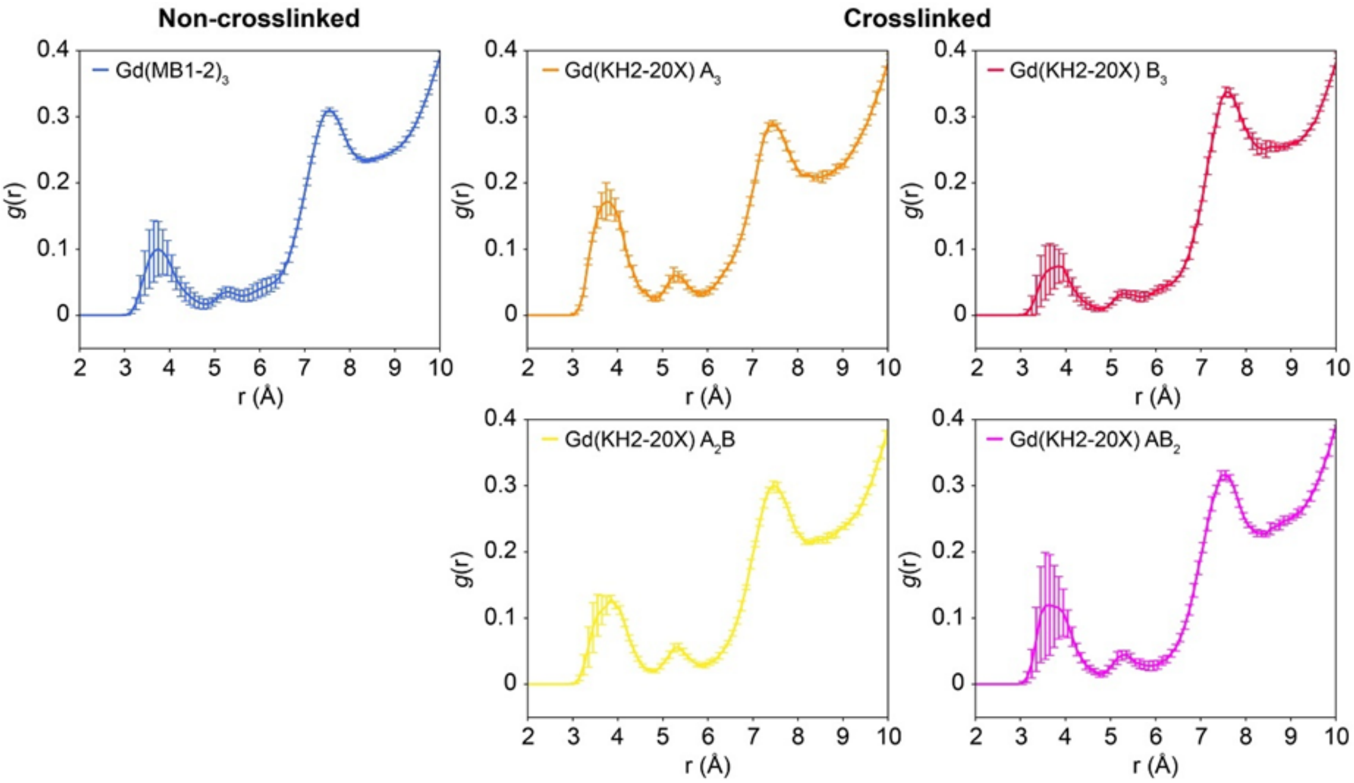

Figure S21: Radial distribution function (RDF) of water hydrogen atoms (H_{Wat}) from Gd(III) in noncross-linked $Gd(MB1\text{-}2)_3$ and cross-linked Gd(KH2-20X) systems. The thick lines indicate the average *g*(r) at each radial distance, while the error bars show the standard deviation across replicate MD simulations. RDFs were calculated by averaging over all frames and independent trajectories for each system.

Figure S22: Comparison of second-sphere water probability

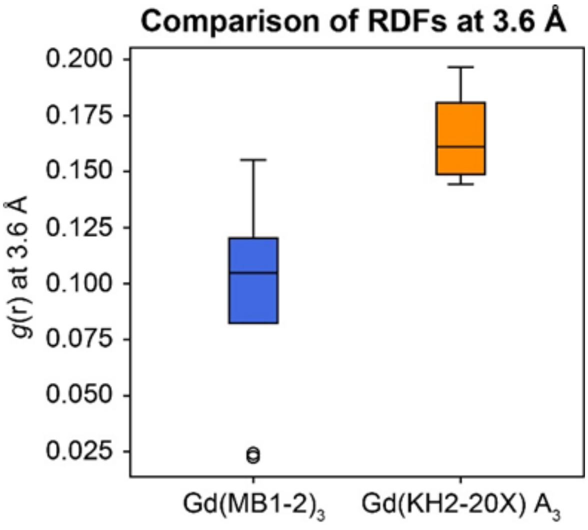

Figure S22: Statistical comparison of the probability of finding water near the metal ion at r = 3.6 Å in Gd(MB1-2)$_3$ versus Gd(KH2-20X) A$_3$ systems. Both a two-sample Student's t-test ($t = -3.53$, $p = 5.5 \times 10^{-3}$) and the non-parametric Mann–Whitney U test ($U = 5.0$, $p = 1.27 \times 10^{-2}$) revealed a statistically significant difference between the two systems at this distance ($p < 0.05$). In the accompanying boxplots, the median is indicated by a black line within each box, and outliers are displayed as black circles outside the whiskers.

Figure S23 Average water molecules in the SS for Gd(MB1-2)$_3$ and Gd(KH2-20X)

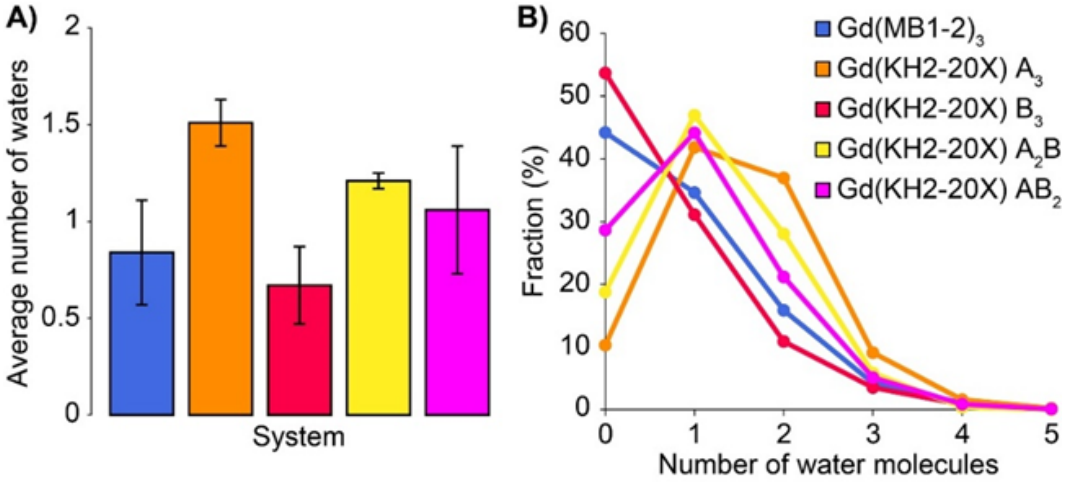

Figure S23: A) Average number of water molecules in the second coordination sphere around Gd(III) (defined as H_{Wat}-Gd(III) distance ≤ 4.5 Å) in Gd(MB1-2)$_3$ and Gd(KH2-20X) systems, calculated from multiple MD simulation replicas. Error bars represent the standard deviation across the replicas. B) Normalized fraction of water molecules found in the second-sphere around Gd(III) during MD simulations for each system.

Table S4: Longitudinal (r_1) and transverse (r_2) relaxivities for Gd(MB1-2)$_3$ and Gd(KH2-20X) at 60 MHz and 298 K, in the absence and presence of 0.6 mM Human Serum Albumin (HSA).

Peptide Sample	r_1 (mM s^{-1})	r_2 (mM s^{-1})
Gd(MB1-2)$_3$	15.3 ± 0.6	20.7 ± 0.1
Gd(MB1-2)$_3$ + HSA	17.4	18.3
Gd(MB1-2)$_3$ + HSA, after 3 days	13.7	14.4
Gd(KH2 -20X)	19.6 ± 0.2	37.4 ± 0.4
Gd(KH2 -20X) + HSA	20.6	40.2
Gd(KH2 -20X) + HSA, after 3 days	20.0	36.0

References

(1) Chan, W.C.; White, P.D. *Fmoc Solid Phase Peptide Synthesis: A Practical Approach*; New York; Oxford University Press; 2000.

(2) Peacock, A.F.A.; Bullen, G.A.; Gethings, L.A.; Williams, J.P.; Kriel, F.H.; Coates, J. Gold-Phosphine Binding to *de Novo* Designed Coiled Coil Peptides. *Journal of Inorganic Biochemistry* 2012, *117* 298–305. DOI: 10.1016/j.jinorgbio.2012.05.010.

(3) Wang, C.; Lai, W.; Yu, F.; Zhang, T.; Lu, L.; Jiang, X.; Zhang, Z.; Xu, X.; Bai, Y.; Jiang, S.; Liu, K. De Novo Design of Isopeptide Bond-Tethered Triple-Stranded Coiled Coils with Exceptional Resistance to Unfolding and Proteolysis: Implication for Developing Antiviral Therapeutics. *Chemical Science* 2015, *6* (11), 6505–6509. DOI: 10.1039/C5SC02220G.

(4) Wang, C.; Li, X.; Yu, F.; Lu, L.; Jiang, X.; Xu, X.; Wang, H.; Lai, W.; Zhang, T.; Zhang, Z.; Ye, L.; Jiang, S.; Liu, K. Site-Specific Isopeptide Bridge Tethering of Chimeric Gp41 N-Terminal Heptad Repeat Helical Trimers for the Treatment of HIV-1 Infection. *Scientific Reports* 2016, *6* (1), 32161. DOI: 10.1038/srep32161.

(5) Barge, A.; Cravotto, G.; Gianolio, E.; Fedeli, F. How to Determine Free Gd and Free Ligand in Solution of Gd Chelates. A Technical Note. *Contrast Media & Molecular Imaging* 2006, *1* (5), 184–188. DOI: 10.1002/cmmi.110.

(6) Vogel, A.I.; Jeffery, G.H. *Vogel's Textbook of Quantitative Chemical Analysis*; Harlow, Essex, England : New York; Longman Scientific & Technical ; Wiley; 1989.

(7) Shah, A.; Taylor, M.J.; Molinaro, G.; Anbu, S.; Verdu, M.; Jennings, L.; Mikulska, I.; Diaz-Moreno, S.; El Mkami, H.; Smith, G.M.; Britton, M.M.; Lovett, J.E.; Peacock, A.F.A. Design of the Elusive Proteinaceous Oxygen Donor Copper Site Suggests a Promising Future for Copper for MRI Contrast Agents. *Proceedings of the National Academy of Sciences* 2023, *120* (27), e2219036120. DOI: 10.1073/pnas.2219036120.

(8) Evans, D.F. 400. The Determination of the Paramagnetic Susceptibility of Substances in Solution by Nuclear Magnetic Resonance. *Journal of the Chemical Society (Resumed)* 1959, (0), 2003–2005. DOI: 10.1039/JR9590002003.

(9) Myers, J.; Pace, C.; Scholtz, J. Helix Propensities Are Identical in Proteins and Peptides. *Biochemistry* 1997, *36* (36), 10923–10929. DOI: 10.1021/bi9707180.

(10) Mattocks, J.A.; Tirsch, J.L.; Cotruvo, J.A. Chapter Two - Determination of Affinities of Lanthanide-Binding Proteins Using Chelator-Buffered Titrations. *Methods in Enzymology* 2021, *651* 23–61. DOI: 10.1016/bs.mie.2021.01.044.

(11) MATLAB. 2022, .

(12) Jumper, J.; Evans, R.; Pritzel, A.; Green, T.; Figurnov, M.; Ronneberger, O.; Tunyasuvunakool, K.; Bates, R.; Žídek, A.; Potapenko, A.; Bridgland, A.; Meyer, C.; Kohl, S.A.A.; Ballard, A.J.; Cowie, A.; Romera-Paredes, B.; Nikolov, S.; Jain, R.; Adler, J.; Back, T.; Petersen, S.; Reiman, D.; Clancy, E.; Zielinski, M.; Steinegger, M.; Pacholska, M.; Berghammer, T.; Bodenstein, S.; Silver, D.; Vinyals, O.; Senior, A.W.; Kavukcuoglu, K.; Kohli, P.; Hassabis, D. Highly Accurate Protein Structure Prediction with AlphaFold. *Nature* 2021, *596* (7873), 583–589. DOI: 10.1038/s41586-021-03819-2.

(13) Evans, R.; O'Neill, M.; Pritzel, A.; Antropova, N.; Senior, A.; Green, T.; Žídek, A.; Bates, R.; Blackwell, S.; Yim, J.; Ronneberger, O.; Bodenstein, S.; Zielinski, M.; Bridgland, A.; Potapenko, A.; Cowie, A.; Tunyasuvunakool, K.; Jain, R.; Clancy, E.; Kohli, P.; Jumper, J.; Hassabis, D. Protein Complex Prediction with AlphaFold-Multimer. 2021, . DOI: 10.1101/2021.10.04.463034.

(14) Mirdita, M.; Schütze, K.; Moriwaki, Y.; Heo, L.; Ovchinnikov, S.; Steinegger, M. ColabFold: Making Protein Folding Accessible to All. *Nature Methods* 2022, *19* (6), 679–682. DOI: 10.1038/s41592-022-01488-1.

(15) Berwick, M.R.; Lewis, D.J.; Jones, A.W.; Parslow, R.A.; Dafforn, T.R.; Cooper, H.J.; Wilkie, J.W.; Pikramenou, Z.; Britton, M.M.; Peacock, A.F.A. De Novo Design of Ln(III) Coiled Coils for Imaging Applications. *Journal of the American Chemical Society* 2014, *136* (4), 1166–1169. DOI: 10.1021/ja408741h.

(16) Dürr, S.L.; Levy, A.; Rothlisberger, U. Metal3D: A General Deep Learning Framework for Accurate Metal Ion Location Prediction in Proteins. *Nature Communications* 2023, *14* (1), 2713. DOI: 10.1038/s41467-023-37870-6.
(17) Maier, J.A.; Martinez, C.; Kasavajhala, K.; Wickstrom, L.; Hauser, K.E.; Simmerling, C. ff14SB: Improving the Accuracy of Protein Side Chain and Backbone Parameters from ff99SB. *Journal of Chemical Theory and Computation* 2015, *11* (8), 3696–3713. DOI: 10.1021/acs.jctc.5b00255.
(18) Frisch, M.J.; Trucks, G.W. Gaussian 16. 2016, .
(19) Jorgensen, W.L.; Chandrasekhar, J.; Madura, J.D.; Impey, R.W.; Klein, M.L. Comparison of Simple Potential Functions for Simulating Liquid Water. *The Journal of Chemical Physics* 1983, *79* (2), 926–935. DOI: 10.1063/1.445869.
(20) Li, P.; Song, L.F.; Merz, K.M.Jr. Parameterization of Highly Charged Metal Ions Using the 12-6-4 LJ-Type Nonbonded Model in Explicit Water. *The Journal of Physical Chemistry B* 2015, *119* (3), 883–895. DOI: 10.1021/jp505875v.
(21) Ryckaert, J.-P.; Ciccotti, G.; Berendsen, H.J.C. Numerical Integration of the Cartesian Equations of Motion of a System with Constraints: Molecular Dynamics of *n*-Alkanes. *Journal of Computational Physics* 1977, *23* (3), 327–341. DOI: 10.1016/0021-9991(77)90098-5.
(22) https://github.com/sulfierry/free_energy_landscape
(23) The PyMOL Molecular Graphics System, Version 2.5.4, Schrödinger, LLC. .
